# Measurements of Antibacterial Activity of Seed Crude Extracts in Cultivated Rice and Wild *Oryza* Species

**DOI:** 10.1186/s12284-022-00610-3

**Published:** 2022-12-13

**Authors:** Yuri Yoshida, Misuzu Nosaka-T, Takanori Yoshikawa, Yutaka Sato

**Affiliations:** 1 National Institute of Genetics, Shizuoka, Japan; 2 Department of Genetics, School of Life Science, Sokendai (Graduate University for Advanced Studies), Shizuoka, Japan; 3 Graduate School of Agriculture, Kyoto University, Kyoto, 606-8502 Japan

**Keywords:** Rice, *Oryza*, Core collection, Wild rice, Seed, Antibacterial activity, GWAS

## Abstract

Seeds are continuously exposed to a wide variety of microorganisms in the soil. In addition, seeds contain large amounts of carbon and nitrogen sources that support initial growth after germination. Thus, seeds in the soil can easily promote microbial growth, and seeds are susceptible to decay. Therefore, seed defense against microorganisms is important for plant survival. Seed-microbe interactions are also important issues from the perspective of food production, in seed quality and shelf life. However, seed-microbe interactions remain largely unexplored. In this study, we established a simple and rapid assay system for the antibacterial activity of rice seed crude extracts by colorimetric quantification methods by the reduction of tetrazolium compound. Using this experimental system, the diversity of effects of rice seed extracts on microbial growth was analyzed using *Escherichia coli* as a bacterial model. We used collections of cultivated rice, comprising 50 accessions of Japanese landraces, 52 accessions of world rice core collections, and of 30 wild *Oryza* accessions. Furthermore, we attempted to find genetic factors responsible for the diversity by genome-wide association analysis. Our results demonstrate that this experimental system can easily analyze the effects of seed extracts on bacterial growth. It also suggests that there are various compounds in rice seeds that affect microbial growth. Overall, this experimental system can be used to clarify the chemical entities and genetic control of seed-microbe interactions and will open the door for understanding the diverse seed-microbe interactions through metabolites.

**Supplementary Information:**

The online version contains supplementary material available at 10.1186/s12284-022-00610-3.

## Background

Plants are exposed to a wide variety of microorganisms throughout their life cycle and constantly interact with these microorganisms. Among the microorganisms that interact with plants, symbiotic microorganisms such as mycorrhizal fungi absorb nutrients from the soil and supply them to plants, thereby positively affecting plant growth. On the other hand, infection and proliferation by microorganisms can negatively affect plant growth. It is known that plants prevent infection by releasing a group of compounds with antimicrobial activity against pathogenic microorganisms (Bednarek and Osbourn 2009). Many of these compound groups are secondary metabolites, and plants have acquired the ability to produce a variety of antimicrobial secondary metabolites in their interactions with microorganisms. Phytoanticipin and phytoalexin are antimicrobial compounds that are synthesized inducibly or not, respectively, and the same compound may act as a phytoanticipin or phytoalexin depending on plant type and organs (VanEtten et al. 1994; Morrissey and Osbourn 1999; Jeandet 2018).

Plant chemical defense using these antimicrobial secondary metabolites has been extensively studied, and to date, numerous antimicrobial secondary metabolites have been found in various plant species. Saponins are well known as phytoanticipins, with α-tomatin in tomatoes and avenacin in oats (Oros and Kállai 2019). For phytoalexins, their bioactivity, synthetic pathways, and induction mechanisms have been analyzed in various plant species (Liu et al. 2010; Schmelz et al. 2011; Ube et al. 2021).

Seed defense against microorganisms is especially important because seeds store sufficient nutrients for seedling establishment, and these seed reserves are also a source of nutrients for microorganisms. Seed defense mechanisms against soil microorganisms can be broadly classified into physical, biochemical, and chemical defenses (Dalling et al. 2020). In physical defense, the seed coat and pericarp serve as a physical barrier between the seed, including the embryo, and its external environment, and suppress microbial invasion (Gergerich and Dolja 2006). Polyphenol oxidase (PPO) is a known defense enzyme involved in biochemical defense, which accumulates in wild oat and wheat glumes (Jerkovic et al. 2010; Fuerst et al. 2011, 2018). PPO catalyzes the conversion of phenolic compounds to quinones, and the quinones may protect seeds from microbes by damaging microbial cell walls (Baltas et al. 2017; Alibi et al. 2021).

Various antimicrobial secondary metabolites identified in studies with rhizosphere and aboveground parts of plants (leaves) have also been identified in seeds (Dalling et al. 2011; Ben-Abu and Itsko 2021; Ishihara 2021). This suggests that chemical defense against microorganisms via antimicrobial secondary metabolites may also operate in seeds.

Momilactones, well-known antimicrobial secondary metabolites of rice, have been studied not only for their functions as phytoalexins but also for their biosynthetic pathways and diversity (Kato et al. 1973; Izawa and Shimamoto 1996; Cartwright et al. 1981; Peters 2006; Schmelz et al. 2014). More than 20 phytoalexins have been reported to accumulate in rice (Kodama et al. 1992; Yamane 2013; Park et al. 2013, 2014; Ishihara et al. 2008; Morimoto et al. 2018; Kariya et al. 2020). The World Rice Core Collection (WRC) (Kojima et al. 2005) and the Rice Core Collection of Japanese Landraces (JRC) (Ebana et al. 2008), which are provided by the National Agriculture and Food Research Organization (NARO, Japan), cover a wide range of genetic diversity of landraces in rice, and they are useful material for the isolation of related quantitative trait loci by GWAS (genome-wide association studies) and for the elucidation of loci associated with phenotypic diversity (Tanaka et al. 2020, 2021). Analysis using the WRC revealed that the amount and type of phytoalexins induced in stress-treated leaves differ between accessions, and novel antimicrobial secondary metabolites have been found in the leaves of several accessions (Kariya et al. 2019, 2020; Murata et al. 2020). In addition, Friedman (2013) reported that different rice secondary metabolites are produced in specific organs and tissues.

The genus *Oryza* consists of 23 species, of which 2 are cultivated and the remaining 21 are wild species, with 11 genome types (AA, BB, CC, BBCC, CCDD, EE, FF, GG, KKLL, HHJJ, HHKK) (Lu et al. 2009; Jacquemin et al. 2013; Nonomura et al. 2010; Sato et al. 2021; Kajiya-Kanegae et al. 2021). Remarkable differences have been observed between metabolite diversity in wild and cultivated *Oryza* species (Atwell et al. 2014). Among the wild *Oryza* species, momilactones are produced in species with AA genome (*O. barthii*, *O. glumaepatula*, *O. meridionalis* and *O. rufipogon*) and BB genome (*O. punctata*), but not in *O. brachyantha* with FF genome, and phytocasans are produced in species with AA genome but not in species with BB genome or FF genome (Miyamoto et al. 2016). These findings suggest that the composition and the amount of secondary metabolites produced by wild and cultivated *Oryza* species are different. Therefore, wild *Oryza* species may accumulate antimicrobial secondary metabolites that are not present in cultivated species, and wild *Oryza* species are attractive materials to search for novel and useful secondary metabolites.

Disk diffusion method is the most common method for measuring the antimicrobial activity. However, quantification of antimicrobial activity is difficult with this method. To overcome these issues, we performed colorimetric assays using tetrazolium salts, which are widely used to measure cell growth, mainly for mammalian cells but also for bacteria (Eloff 1998; Tsukatani et al. 2009; Haase et al. 2017; Grela et al. 2015; Benov 2021).

**Fig. 1:**
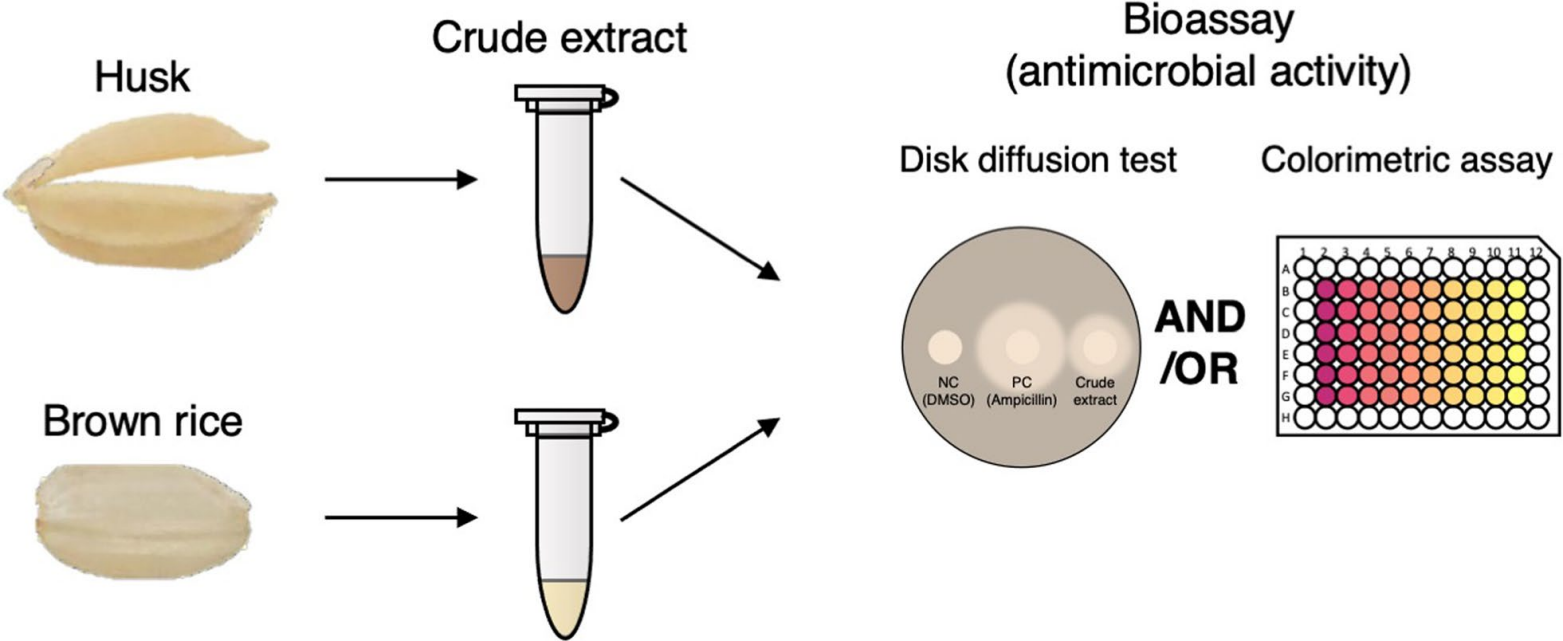
Schematic diagram of the antibacterial activity test of rice seed crude extract

To understand the chemical defense of seeds mediated by antimicrobial secondary metabolites, we developed a simple and rapid assay system to evaluate the antibacterial activity of rice seed crude extracts using disk diffusion and colorimetric quantification methods (Fig. 1). Furthermore, we investigated the diversity of antibacterial activity of rice seeds using the rice core collection of landraces WRC, JRC and wild *Oryza* species, and searched for genetic factors associated with the diversity of antibacterial activity in the landraces using GWAS. Here we demonstrate a colorimetric assay system that can detect antibacterial activity of rice seed crude extracts with higher sensitivity than the disk diffusion method. The results of the measurements of antibacterial activity of rice seeds of landraces and wild *Oryza* species using this assay system suggest that the antibacterial activity differs between accessions and that several types of antibacterial secondary metabolites are extracted with different solvents. Through comparing the antibacterial activity of husk and brown rice extracts, different types and amounts of antibacterial compounds are suggested to accumulate in these organs. Furthermore, GWAS using the antibacterial activity of husk and brown rice extracts measured by our assay system detected several genomic regions related to the diversity of antibacterial activity. The assay system established in this study using diverse genetic resources is expected to serve as a stepping stone to explore seed-microbe interactions and antibacterial secondary metabolites produced by seeds and to contribute to the elucidation of their genetic basis.

## Materials and Methods

### Plant Materials

The seeds of World Rice Core Collection (WRC) (Kojima et al. 2005) and Rice Core Collection of Japanese landraces (JRC) (Ebana et al. 2008) were obtained from the National Agriculture and Food Research Organization Genebank (NARO gene bank, Japan, http://www.gene.affrc.go.jp/index_j.php). The seeds of cultivated rice (*Oryza sativa* L. ‘T65’) and wild *Oryza* species were provided by the National Institute of Genetics (NIG, Japan, https://www.nig.ac.jp/nig/ja/). Plants were grown under natural light condition in a green-house and transplanted to paddy fields at NIG in Mishima, Japan, and the collected seeds were analyzed in this study.

### Preparation of Seed Crude Extracts

The collected seeds were separated into husks and brown rice using testing rice husker (Fujiwara factory, Tokyo, Japan). The weight of husks and brown rice in 10 grains were measured (Additional file 1: Table S1). Because the weight of husks and brown rice in one accession are constant among grains, we use fix number of grains in all experiments so that weight of tissues used for extraction become constant in replicated experiments. To prepare husk and brown rice crude extracts, rice husks of 20 grains and 10 grains of brown rice were pulverized in 5 mL microtubes, respectively, by a multi-beads shocker (Yasui Kikai Corporation, Japan). Powdered samples in 5 mL microtubes were immersed in 4 mL of four different solvents (80% MeOH, diethyl ether, acetone, and sterilized water, respectively) under two different temperature (room temperature for diethyl ether and acetone extraction, 80 °C for 80% MeOH and sterilized water extraction) for 24 h. For brown rice crude extracts, diethyl ether was not used as solvent. After filter-sterilization using 0.22 μm membrane filters (Membrane Solution Limited, USA), solvents were removed by a centrifugal concentrator and each crude extracts was stored at − 20 °C for further analysis.

### Antibacterial Activity Test by Disk Diffusion Method

Antibacterial activity of the rice husk crude extract was determined against *Eschelicia coli* DH5α by disk diffusion method according to Fukuta et al. (2007), with some modifications. Muller-Hinton (MH) broth medium was used to grow the bacteria. Single colony of *E. coli* were inoculated to 4 mL of MH broth and incubated at 37 °C for overnight at 180 rpm in the shaker. Cell density of overnight cultures of *E. coli* was determined by spectrophotometer (Bio-Rad Laboratories, USA) and suspended in 0.9% sterilized saline to 2.0 × 10^5^ cell/mL. 500 μL of the bacteria suspension was spread evenly on each MH agar plate (9 cm diameter). Crude extracts of 20 grains of rice husk (solvents: MeOH, acetone, and sterilized water, respectively) and 80 grains of rice husk (solvent: diethyl ether) were dried and dissolved in 30 μL of 100% dimethyl sulfoxide (DMSO). The crude extracts of 20 grains of rice husk, and only diethyl ether crude extracts were equal to 80 grains. Sterilized paper disks (6 mm diameter, ADVANTEC) were impregnated by 30 μL of each extract dissolved in 100% DMSO and laid on the surface of MH agar plates. Ampicillin (10 μg/mL) and 100% DMSO were used as positive and negative control, respectively. The plates were incubated for 24 h at 37 °C and inhibition zone was measured.

### Antibacterial Activity Test by Colorimetric Quantification Assay

Antibacterial activity of rice husk and brown rice crude extracts was determined by microtiter plate bioassay method using the tetrazolium compound [3-(4,5-dimethylthiazol-2-yl)-5-(3-carboxymethoxyphenyl)-2-(4-sulfophenyl)-2H-tetrazolium, inner salt (MTS)] as a color indicator (CellTiter 96® AQueous One Solution Cell Proliferation Assay, Promega, USA) according to the manufacture’s instructions. Relative growth rate of *E. coli*, which represents the antibacterial activity of the sample extracts, were measured for each sample extracts. DMSO and antibiotics (ampicillin and kanamycin) are negative and positive controls of antibacterial activity.

### Concentration-Dependent Growth Inhibitory Effect of Seed Crude Extracts on *Escherichia coli*

Rice husk crude extracts were prepared from accessions that showed antibacterial activity by the disk diffusion method and their concentration-dependent growth inhibitory effect on *E. coli* was investigated. To determine concentration-dependent effects, rice husk crude extracts were prepared from 20 grains of rice husk. The extracts were dried and dissolved in 40 μL DMSO for 80% MeOH extracts, 20 μL DMSO for acetone extracts and 10 μL DMSO for sterilized water extracts, respectively, as stock solutions. Brown rice crude extracts were prepared from 10 grains of brown rice. The extracts were dried and dissolved in 10 μL of DMSO as stock solution.

20 μL rice crude extracts were transferred to the first well of 96-well plate and serial dilution was done by transferring 10 μL mixture via multichannel pipette. Then, 180 μL of MH broth was poured into each of the 96 wells of the assay plate. Bacterial cells were added at a density of 4.0 × 10^6^ cells/mL (10 μL) in 96 wells. Ampicillin and kanamycin were used as a positive control. A growth control with 10 μL suspension solvent and blank control without bacteria were also loaded in the assay plate. The assay plates were incubated at 37 °C for 5 h in a humid environment. After incubation, 40 μL of MTS solution was added to each well and mixed. The plates were then centrifuged at 600 × *g* for 5 min and 100 μL of the supernatant was transferred to a new 96-well plate and the absorbance at 450 nm at 0 h was measured using a microplate reader. The assay plates were incubated again at 37 °C for 4 h. After the second incubation, the plate proceeded to centrifugation at 600 × *g* for 5 min and 100 μL of the supernatant was transferred to a new plate and the absorbance of after 4 h was measured. Relative growth rates of bacteria treated with antibiotics and/or crude extracts were calculated by taking the increase in absorbance of the growth control wells as 1. Relative growth rate was calculated as the change in absorbance after 4 h in wells treated with antibiotics and/or seed crude extract. The increase in absorbance after 4 h in wells of the growth control was set as 100%.

### Evaluation of Antibacterial Activity of Seeds of Cultivated and Wild Rice Core Collections

Antibacterial activity assays for cultivated and wild *Oryza* species were analyzed in the same way using certain dilutions of each crude extract as described above. Based on the results of the measurements of concentration-dependent antibacterial activity, the dilution factors that showed stronger activity than the value of 50% of maximum inhibitory concentration (IC50) in the rice husk and brown rice crude extracts using three different solvents were used in the subsequent assays.

### Construction of Genetic Map

Sequence data for JRC and WRC derived from the previous study in Tanaka et al. (2020) and Tanaka et al. (2021) were downloaded from DNA Data Bank of Japan Sequence Read Archive. Trimming of raw paired-end reads and subsequent mapping against Os-Nipponbare-Reference-IRGSP-1.0 (Kawahara et al. 2013) were performed using Galaxy/NAAC, a web-based platform for a bioinformatics analysis (https://galaxy.dna.affrc.go.jp/nias/static/register_en.html). “Trimmomatic” function (Galaxy Version 0.36.3) and “BWA mapping Illumina” workflow were used for the removal of low quality reads and mapping against the reference sequence, respectively. Obtained bam files were used to create gVCF files using GATK (ver.4.2.2.0) HaplotypeCaller (McKenna et al. 2010; DePristo et al. 2011), and then gVCF files were consolidated with CombineGVCFs. Variants called with GenotypeGVCFs were then filtered using “view” function in bcftools (Li 2011) with the following parameters: -m2 -M2 -g ^het --output-type z --exclude-uncalled -e "MAF < 0.05 || N_MISSING > 17 || QD < 2.0 || QUAL < 30.0 || SOR > 3.0 || FS > 60.0 || MQ < 40.0 || MQRankSum < -12.5 || ReadPosRankSum < -8.0" to obtain 1,213,105 variants with the maximum minor allele frequency of 5% and the minimum call rate of 85% without heterozygous haplotype.

### GWAS

The Weighted Mixed Linear Model in TASSEL (Bradbury et al. 2007) was used for the GWAS with the option of Re-estimate after each marker. The visualization of manhattan plots and qq plots were performed with R package qqman (Turner 2014). The false discovery rate (FDR) was calculated with Benjamini–Hochberg procedure (Benjamini and Hochberg 1995), and SNPs (Single nucleotide polymorphisms) with FDR of less than 5% were considered as significant association. Genomic linkage disequilibrium (LD) decay was estimated based on the coefficients of determination (r^2^) between all pairs of loci using PopLDdecay (ver. 3.41) (Zhang et al. 2019) in a 2000 kb distance, and it was considered that LD was decayed at 940 kb in our genetic map since r^2^ was less than 0.25 at this genetic distance. Therefore, subsequent LD analysis was performed within this distance using R package LDheatmap (Shin et al. 2006), and the continuous markers with r^2^ more than 0.5 were considered to belong to the same LD block.

## Results

### Detection of Antibacterial Activity of Rice Seeds Crude Extracts by Disk Diffusion Methods

**Fig. 2:**
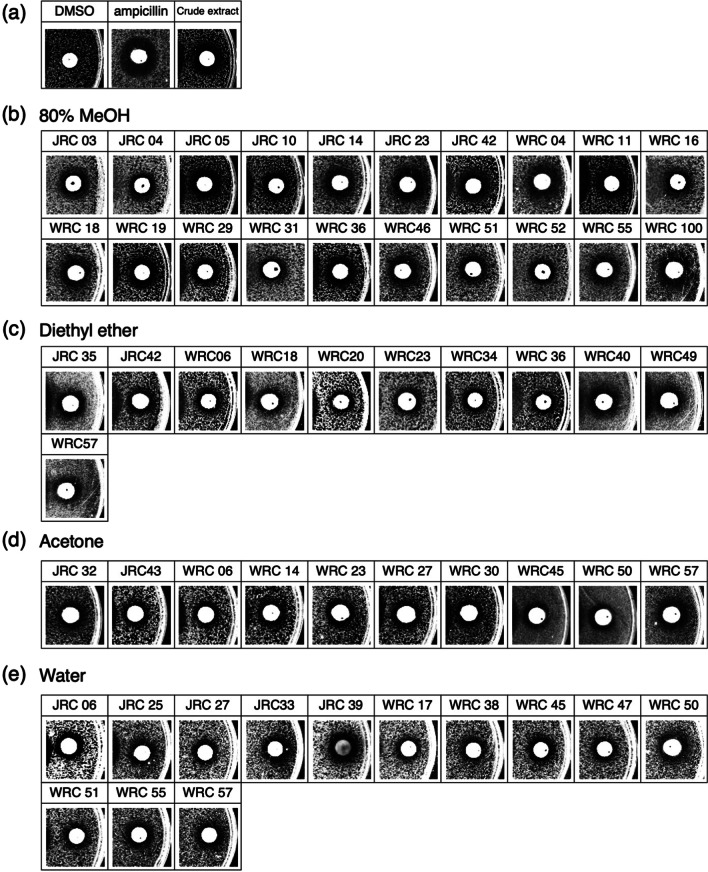
Antibacterial activity test of rice husk crude extracts of landraces using disk diffusion method. **a** Inhibition zone detected by disk diffusion method using husk crude extract of cultivated rice (T65; Taichung) extracted with 80% MeOH. DMSO and ampicillin indicate negative and positive controls, respectively. **b**–**e** Inhibition zone detected by disk diffusion method using husk crude extract of accessions of Rice Core Collection of Japanese Landraces (JRC) and World Rice Core Collection (WRC) with antibacterial activities. Solvent of extraction were 80% MeOH (**b**), diethyl ether (**c**), acetone (**d**), and sterilized water (**e**), respectively

In order to elucidate the defense mechanism against microorganisms, we aimed to establish assay systems for the antibacterial activity of rice seeds (Fig. 1). First, a crude extract prepared in 80% MeOH from the husks of cultivated rice T65 was used to determine the antibacterial activity against *Escherichia coli* (*E. coli*) (Fig. 2a). We adopted the disk diffusion method where growth inhibition circles were formed around paper disks containing antibacterial substances. Ampicillin, a commonly used antibiotic, was selected as a positive control for antibacterial activity. Growth inhibition circles were formed around discs containing ampicillin, and similarly, growth inhibition circles were observed around discs containing rice husk crude extract. From this result, we concluded that the disc diffusion method can detect antibacterial activity derived from rice husks.

Next, the antibacterial activity of rice husk crude extract was analyzed by the disk diffusion method in 52 cultivars from the World Rice Core Collection (WRC) (Kojima et al. 2005) and 50 cultivars from the Rice Core Collection of Japanese Landraces (JRC) (Ebana et al. 2008). The crude extracts used to detect antibacterial activity were prepared from 20 seeds of rice husks, and 80% MeOH, diethyl ether, acetone, and water were used as extraction solvents. The diethyl ether crude extract was prepared from 80 seed husks.

**Fig. 3:**
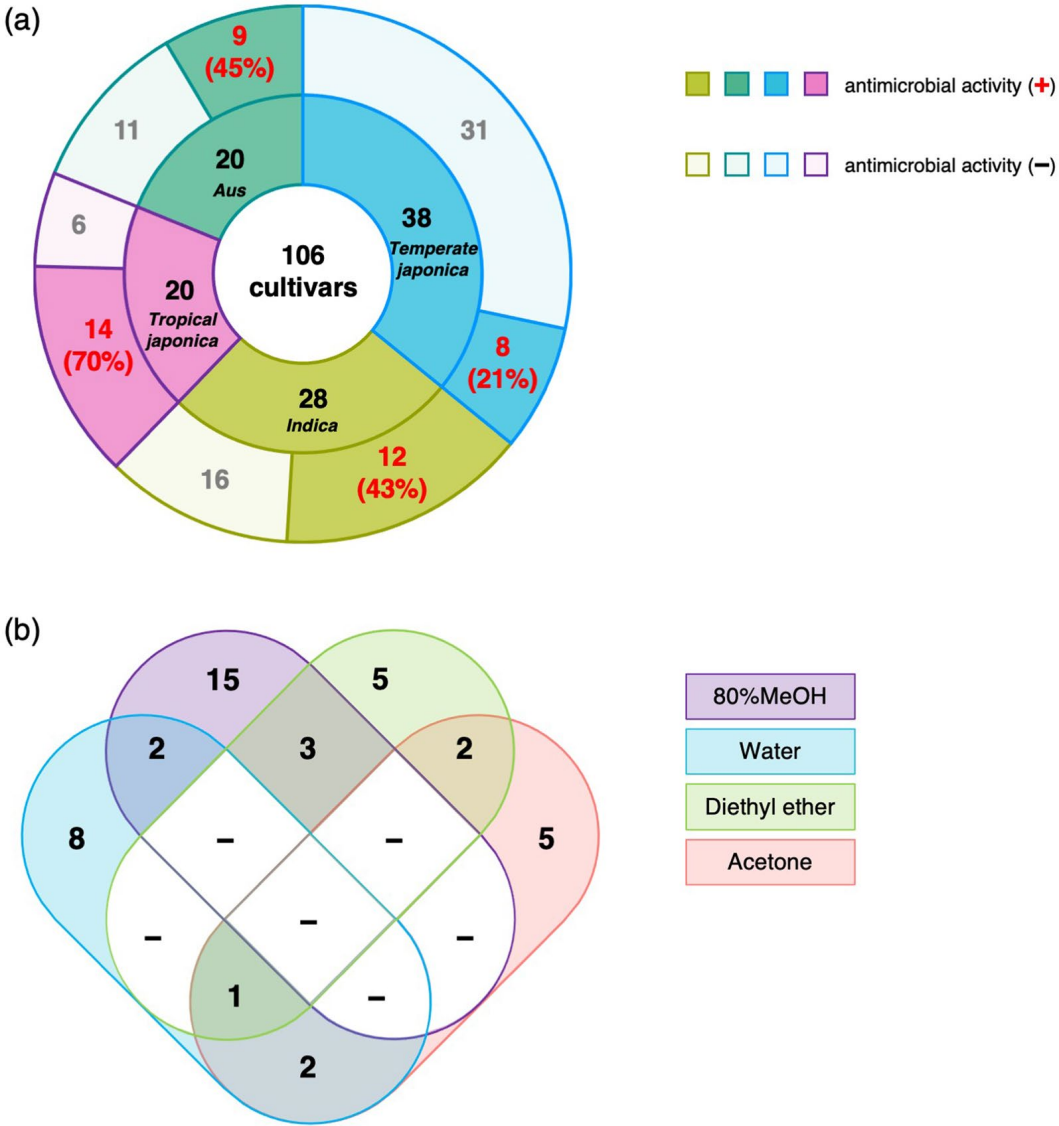
Classification of accessions with antibacterial activity in the husk crude extracts of cultivated rice **a** Proportions of accessions with antibacterial activity in each subspecies of cultivated rice. **b** A Venn diagram showing the number of accessions which showed antibacterial activity in extracts using one or more solvents in cultivated rice

Of a total of 106 cultivars tested, 43 showed antibacterial activity in extracts prepared from any of the solvents (Fig. 2b–e, Tables 1, 2). The cultivated rice core collection is classified into four subspecies: *indica*, *aus*, temperate *japonica*, and tropical *japonica* (Tanaka et al. 2020, 2021). Of the 43 cultivars that showed antibacterial activity, 12 (43%), 9 (45%), 8 (21%), and 14 (70%) belonged to *indica*, *aus*, temperate *japonica*, and tropical *japonica*, respectively (Fig. 3a). Extracts from many of the 43 varieties showed antibacterial activity in one of the four solvents (Fig. 3b). On the other hand, extracts from several varieties showed antibacterial activity in two or more solvents. These varieties may contain multiple antibacterial compounds extracted by different solvents (Fig. 3b).

**Table 1:** Results of antimicrobial activity tests using husk crude extracts from accessions in World Rice Core Collection

World rice core collection		Solvents used to prepare husk crude extracts			
Accession #	Subspecies	80% MeOH	Diethyl ether	Acetone	Water
WRC 01	*Temperate japonica*	−	−	−	−
WRC 02	*Aus*	−	−	−	−
WRC 03	*Indica*	−	−	−	−
WRC 04	*Aus*	+	−	−	−
WRC 05	*Indica*	−	−	−	−
WRC 06	*Indica*	−	+	+	−
WRC 07	*Indica*	−	−	−	−
WRC 09	*Indica*	−	−	−	−
WRC 10	*Indica*	−	−	−	−
WRC 11	*Indica*	+	−	−	−
WRC 12	*Indica*	−	−	−	−
WRC 13	*Indica*	−	−	−	−
WRC 14	*Indica*	−	−	+	−
WRC 15	*Indica*	−	−	−	−
WRC 16	*Indica*	+	−	−	−
WRC 17	*Indica*	−	−	−	+
WRC 18	*Indica*	+	+	−	−
WRC 19	*Indica*	+	−	−	−
WRC 20	*Indica*	−	+	−	−
WRC 21	*Indica*	−	−	−	−
WRC 22	*Indica*	−	−	−	−
WRC 23	*Temperate japonica*	−	+	+	−
WRC 24	*Indica*	−	−	−	−
WRC 25	*Aus*	−	−	−	−
WRC 26	*Aus*	−	−	−	−
WRC 27	*Aus*	−	−	+	−
WRC 28	*Aus*	−	−	−	−
WRC 29	*Aus*	+	−	−	−
WRC 30	*Aus*	−	−	+	−
WRC 31	*Aus*	+	−	−	−
WRC 32	*Aus*	−	−	−	−
WRC 33	*Aus*	−	−	−	−
WRC 34	*Aus*	−	+	−	−
WRC 35	*Aus*	−	−	−	−
WRC 36	*Aus*	+	+	−	−
WRC 37	*Aus*	−	−	−	−
WRC 38	*Aus*	−	−	−	+
WRC 39	*Aus*	−	−	−	−
WRC 40	*Aus*	−	+	−	−
WRC 41	*Aus*	−	−	−	−
WRC 42	*Aus*	−	−	−	−
WRC 43	*Temperate japonica*	−	−	−	−
WRC 44	*Indica*	−	−	−	−
WRC 45	*Tropical Japonica*	−	−	+	+
WRC 46	*Tropical Japonica*	+	−	−	−
WRC 47	*Tropical Japonica*	−	−	−	+
WRC 48	*Tropical Japonica*	−	−	−	−
WRC 49	*Tropical Japonica*	−	+	−	−
WRC 51	*Tropical Japonica*	+	−	−	+
WRC 52	*Tropical Japonica*	+	−	−	−
WRC 55	*Tropical Japonica*	+	−	−	+
WRC 57	*Indica*	−	+	+	+

+ and − represent accessions with or without antimicrobial activity

**Table 2:** Results of antimicrobial activity tests using husk crude extracts from accessions in Rice Core Collection of Japanese Landraces

Rice Core Collection of Japanese Landraces		Solvents used to prepare husk crude extracts			
Accession #	Subspecies	80% MeOH	Diethyl ether	Acetone	Water
JRC 01	*Tropical Japonica*	−	−	−	−
JRC 03	*Tropical Japonica*	+	−	−	−
JRC 04	*Tropical Japonica*	+	−	−	−
JRC 05	*Tropical Japonica*	+	−	−	−
JRC 06	*Tropical Japonica*	−	−	−	+
JRC 07	*Tropical Japonica*	−	−	−	−
JRC 08	*Tropical Japonica*	−	−	−	−
JRC 10	*Tropical Japonica*	+	−	−	−
JRC 11	*Tropical Japonica*	−	−	−	−
JRC 12	*Tropical Japonica*	−	−	−	−
JRC 13	*Tropical Japonica*	−	−	−	−
JRC 14	*Tropical Japonica*	+	−	−	−
JRC 17	*Temperate japonica*	−	−	−	−
JRC 18	*Temperate japonica*	−	−	−	−
JRC 19	*Temperate japonica*	−	−	−	−
JRC 20	*Temperate japonica*	−	−	−	−
JRC 21	*Temperate japonica*	−	−	−	−
JRC 22	*Temperate japonica*	−	−	−	−
JRC 23	*Temperate japonica*	+	−	−	−
JRC 24	*Temperate japonica*	−	−	−	−
JRC 25	*Temperate japonica*	−	−	−	+
JRC 26	*Temperate japonica*	−	−	−	−
JRC 27	*Temperate japonica*	−	−	−	+
JRC 28	*Temperate japonica*	−	−	−	−
JRC 29	*Temperate japonica*	−	−	−	−
JRC 30	*Temperate japonica*	−	−	−	−
JRC 31	*Temperate japonica*	−	−	−	−
JRC 32	*Temperate japonica*	−	−	+	−
JRC 33	*Temperate japonica*	−	−	−	+
JRC 34	*Temperate japonica*	−	−	−	−
JRC 35	*Temperate japonica*	−	+	−	−
JRC 36	*Temperate japonica*	−	−	−	−
JRC 37	*Temperate japonica*	−	−	−	−
JRC 38	*Temperate japonica*	−	−	−	−
JRC 39	*Temperate japonica*	−	−	−	+
JRC 40	*Temperate japonica*	−	−	−	−
JRC 41	*Indica*	−	−	−	−
JRC 42	*Indica*	+	+	−	−
JRC 43	*Indica*	−	−	+	−
JRC 44	*Indica*	−	−	−	−
JRC 45	*Temperate japonica*	−	−	−	−
JRC 46	*Temperate japonica*	−	−	−	−
JRC 47	*Temperate japonica*	−	−	−	−
JRC 48	*Temperate japonica*	−	−	−	−
JRC 49	*Temperate japonica*	−	−	−	−
JRC 50	*Temperate japonica*	−	−	−	−
JRC 51	*Temperate japonica*	−	−	−	−
JRC 52	*Temperate japonica*	−	−	−	−
JRC 53	*Temperate japonica*	−	−	−	−
JRC 54	*Temperate japonica*	−	−	−	−

+ and − represent accessions with or without antimicrobial activity

### Quantitative Measurements of Antibacterial Activity of Rice Seed Crude Extracts

**Fig. 4:**
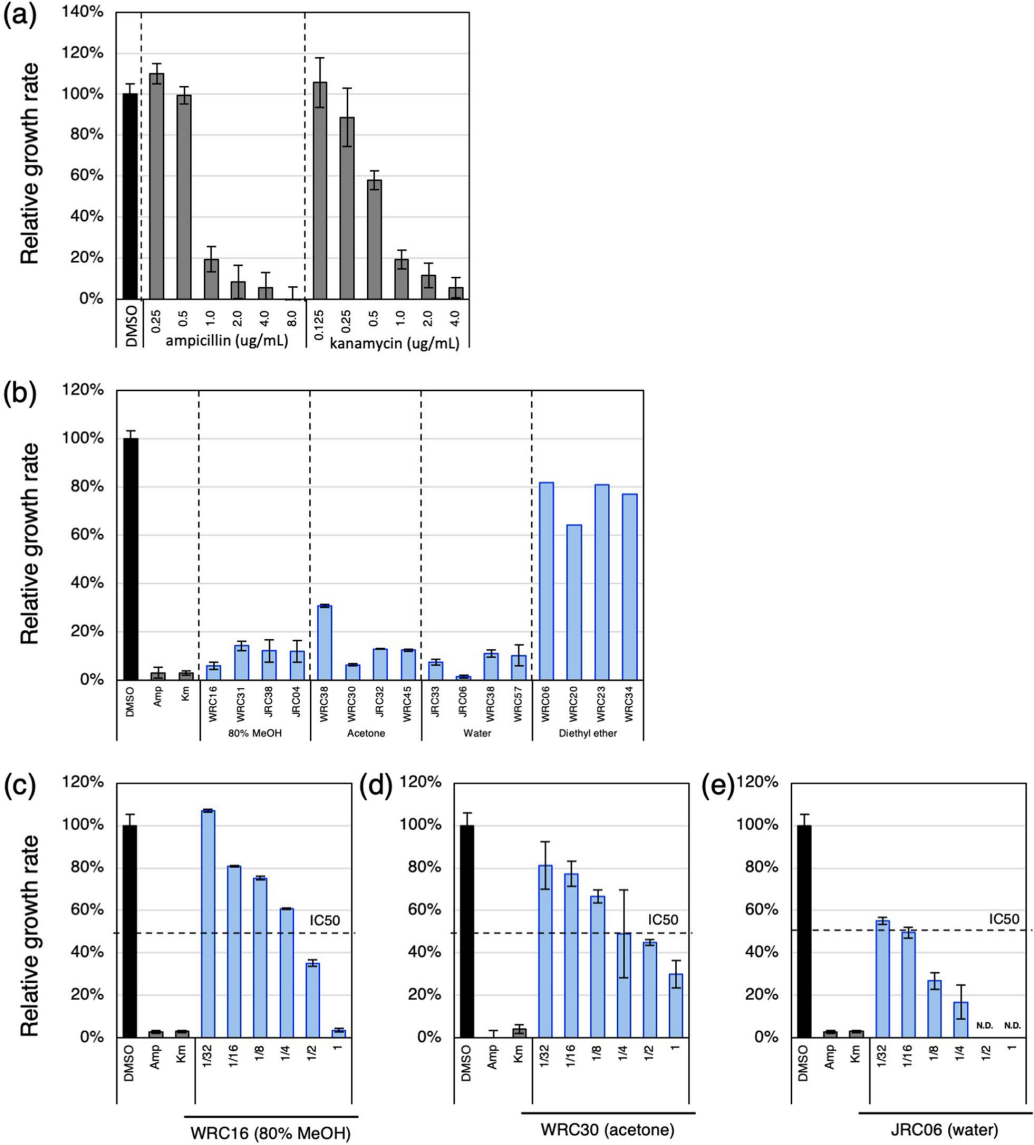
Measurements of antibacterial activity by MTS assay. **a** Dose dependent antibacterial activity of two antibiotics measured by MTS assay. **b** Antibacterial activities measured by MTS assay using four representative accessions in each solvent used for the disk diffusion method. **c**–**e** Dose dependent antibacterial activities measured by MTS assay using extracts from accessions which showed strong antibacterial activities in **b**. x axis in **a** indicates concentration of antibiotics. x axis in **b** indicates the name of the extracts. x axis in **c** indicates concentration of specific extracts. y axis indicates relative growth rate of *E. coli*, which represents the antibacterial activity of the sample extracts. DMSO and antibiotics (ampicillin and kanamycin) are negative and positive controls of antibacterial activity. Values indicate means. Error bars indicate standard deviations. N.D. indicates undetectable level of antibacterial activity. IC50 indicates 50% of maximum inhibitory concentration of ampicillin, which is a positive control. Measurements were triplicated except for diethyl ether extracts

Since it is difficult to quantify antibacterial activity by the disk diffusion method, we developed a simple and rapid assay system for the quantitative measurement of antibacterial activity (Fig. 1). In living cells, the tetrazolium salt, MTS (3-(4,5-dimethylthiazol-2-yl)-5-(3-carboxymethoxyphenyl)-2-(4-sulfophenyl)-2H-tetrazolium, inner salt), is reduced to form formazan. In the MTS assay, the production of formazan by bacterial growth is measured by its specific absorbance and thus effects addition of seed crude extracts on the bacterial growth can be quantified in small-scale culture with high sensitivity. The growth inhibition of bacteria cultured in the presence of the antibiotics ampicillin or kanamycin was examined by the MTS assay and we successfully detected a concentration-dependent decrease in the growth of *E. coli* (Fig. 4a).

Next, we selected four varieties whose antibacterial activity was detected by the disk diffusion method in extracts in each solvent and confirmed the detection of antibacterial activity by the MTS method. As a result, we were able to confirm the growth inhibition effect in extracts from three solvents other than diethyl ether (Fig. 4b). It may be possible that, due to the extremely low solubility of ether extract in DMSO, antibacterial activity is not detected in the MTS assay from diethyl ether extracts. For the above reasons, extracts from three extraction solvents were used for subsequent assays: 80% MeOH, acetone, and water. The dose dependence on bacterial growth inhibition was then examined using husk extracts with the strongest antibacterial activity in each solvent. The half-maximal inhibitory concentrations (IC50) were between two- and three-fold dilutions for 80% MeOH extracts, and around four-fold dilution for acetone and 16-fold dilutions for water extracts, that are equivalent to the amounts of extracts from 2.5, 5, and 2.5 grains dissolved in 10 ul of DMSO, respectively (Fig. 4c–e). Based on these results, we decided to dilute the extracts used in the subsequent assays to fourfold and eightfold for 80% MeOH and acetone, and for water extracts.

### Quantification of Antibacterial Activity in Extracts from Husk and Brown Rice Using Collections of Cultivated Rice

**Fig. 5:**
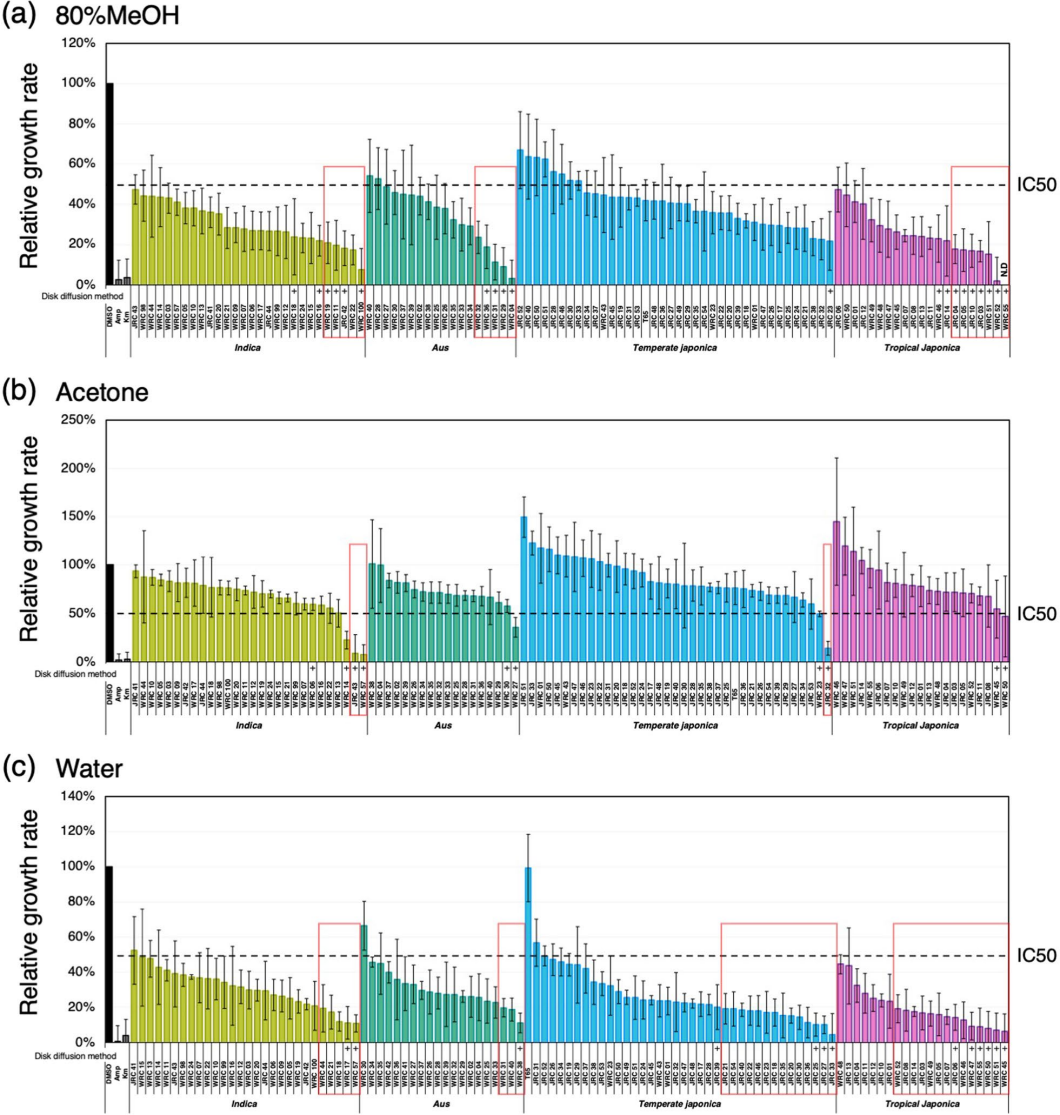
Antibacterial activities of husk crude extracts from accessions in WRC, JRC, and T65 measured by MTS assay. Solvents for extraction were 80% MeOH (**a**), acetone (**b**), and sterilized water (**c**), respectively. x axis indicates the name of the extracts. y axis indicates relative growth rate of *E. coli*, which represents the antibacterial activity of the sample extracts. DMSO and antibiotics (ampicillin and kanamycin) are negative and positive controls of antibacterial activity. Stock solution of extracts were diluted by the concentration of extracts using either 2.5 seeds (**a**, **c**) or 5 seeds (**b**) for extraction and used for the measurement. All measurements were triplicated. Values indicate means. Error bars indicate standard deviation. (+) indicates accessions which showed antibacterial activity in disk diffusion method. Red boxes represent accessions with growth inhibition less than 20% in MTS assay. N.D. indicates undetectable level of antibacterial activity. IC50 indicates 50% of maximum inhibitory concentration of ampicillin, which is a positive control

The MTS assay was used to quantify the antibacterial activity of husk extracts derived from 107 cultivated rice varieties (106 core collection varieties + T65) (Fig. 5a–c). 80% MeOH extracts from most of the 107 varieties inhibited bacterial growth to less than 50%, with extracts from 15 cultivars inhibited to less than 20% (Fig. 5a). Acetone extracts from 7 of the 107 cultivars inhibited growth to less than 50%, with extracts from 3 cultivars inhibited growth to less than 20%. (Fig. 5b). Water extracts from most of the 107 cultivars inhibited growth to less than 50%, with extracts from 36 cultivars inhibited growth to less than 20%. (Fig. 5c). Extracts with antibacterial activities in disk diffusion methods also showed strong antibacterial activities in the MTS assay and the MTS assay detect antibacterial activities in a broader range of cultivars. Thus, the MTS assay seems more sensitive in detecting antibacterial activities.

**Fig. 6:**
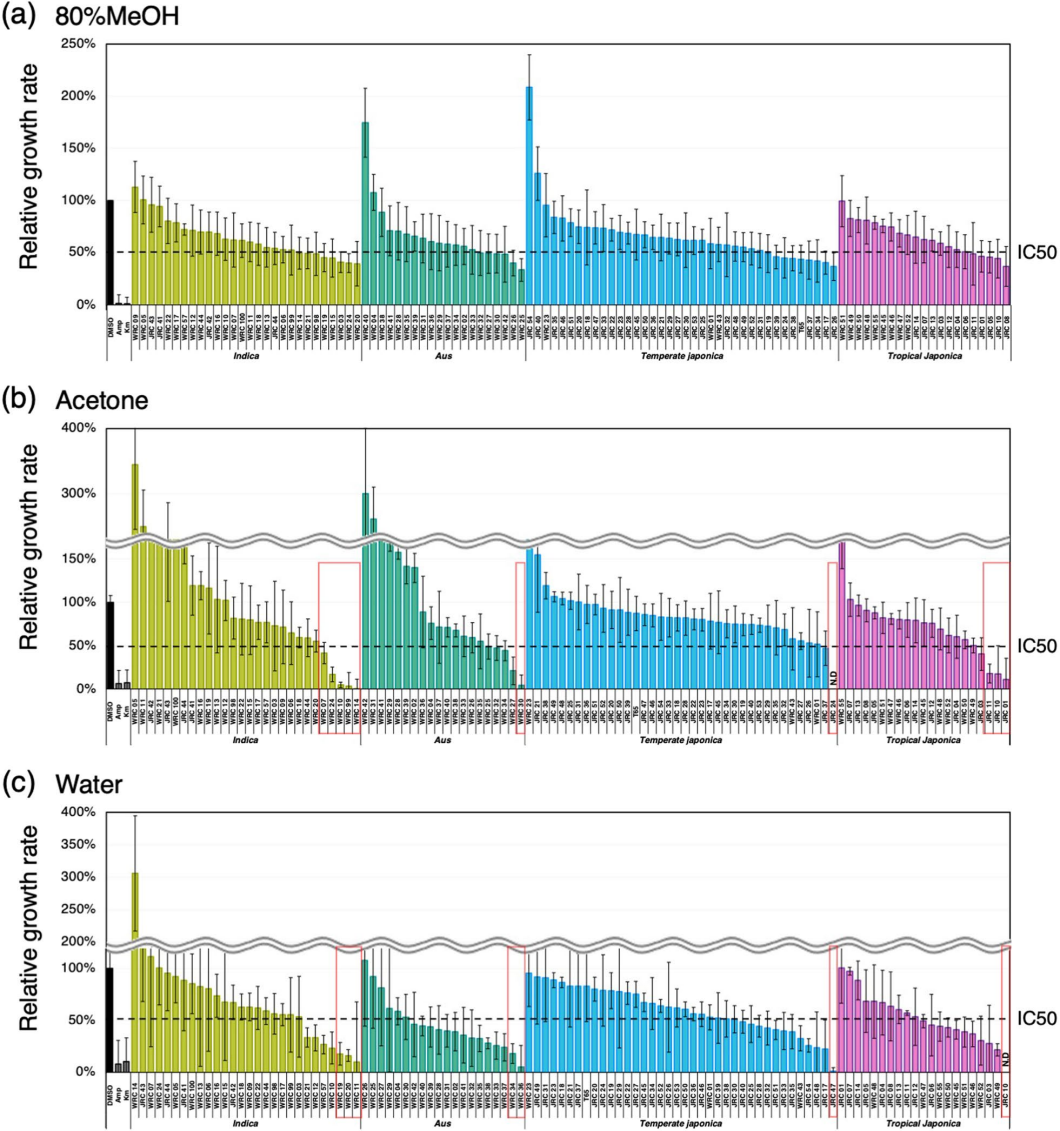
Antibacterial activities of brown rice crude extracts from accessions in WRC, JRC, and T65 measured by MTS assay. Solvents for extraction were 80% MeOH (**a**), acetone (**b**), and sterilized water (**c**), respectively. x axis indicates the name of the extracts. y axis indicates relative growth rate of *E. coli*, which represents the antibacterial activity of the sample extracts. DMSO and antibiotics (ampicillin and kanamycin) are negative and positive controls of antibacterial activity. Stock solution of extracts were diluted by the concentration of extracts using either 2.5 seeds (**a**, **c**) or 5 seeds (**b**) for extraction and used for the measurement. All measurements were triplicated. Values indicate means. Error bars indicate standard deviation. Red boxes represent accessions with growth inhibition less than 20% in MTS assay. N.D. indicates undetectable level of antibacterial activity. IC50 indicates 50% of maximum inhibitory concentration of ampicillin, which is a positive control

The antibacterial activity exhibited by rice seeds is not only derived from the husks but brown rice is also known to possess antimicrobial activity (Gianinetti et al. 2018; Pumirat and Luplertlop 2013). Therefore, we attempted to detect the antibacterial activity of brown rice by MTS assay using 107 cultivated rice varieties (106 core collection varieties + T65). 80% MeOH extracts from 28 of the 107 varieties inhibited bacterial growth to less than 50% and extracts from none of cultivars inhibited to less than 20% (Fig. 6a). Acetone extracts from 17 of the 107 cultivars inhibited growth to less than 50%, with extracts from 9 cultivars inhibited growth to less than 20% (Fig. 6b). Water extracts from 44 of the 107 cultivars inhibited growth to less than 50%, with extracts from 7 cultivars inhibited growth to less than 20% (Fig. 6c). Brown rice extracts from several varieties promoted the growth of bacteria (Fig. 6a–c). Since brown rice is rich in carbon sources such as starch and sugar, it is possible that these compounds in the brown rice extract promoted the growth of bacteria.

### Genome-Wide Association Study to Detect Genetic Factors Causing the Diversity of Bacterial Growth by Extracts from Husk and Brown Rice from Collections of Cultivated Rice

**Fig. 7:**
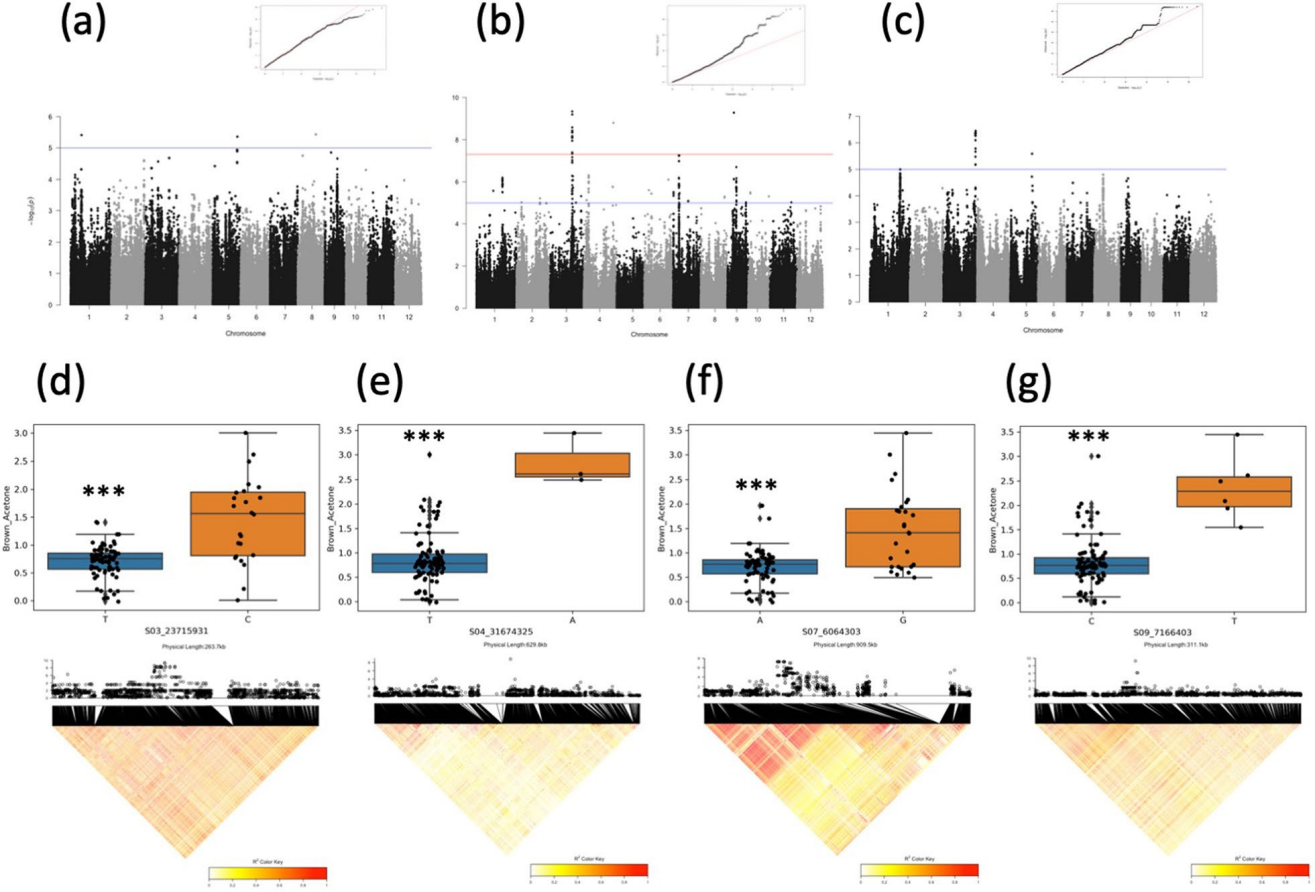
Genome-wide association study using antibacterial activities of rice seed crude extracts from 107 accessions of landraces from WRC and JRC as phenotype data. **a**–**c** Manhattan and Quantile–quantile plots of SNPs associated with antibacterial activity in 80% Methanol extracts from husk (**a**), acetone extracts from brown rice (**b**), and 80% Methanol extracts from husk (**c**). **d**–**g** Upper panels show box plot of relative growth rate of *E. coli* for acetone extracts from brown rice of WRC and JRC accessions with different haplotypes. Lower panels show the LD blocks which carry highly associated SNPs. The accessions were clasified into two haplotypes according to the highest associated SNP in each LD block at chr. 3 (**d**), chr. 4 (**e**), chr. 7 (**f**), and chr. 9 (**g**). In the upper panels, x axis indicates haplotype and y axis indicates relative growth rate of *E. coli*, which represents the antibacterial activity of the sample extracts. ***p < 0.001 (Student’s *t*-test)

Genome-wide association study was conducted to detect genetic factors causing the diversity of bacterial growth by extracts from husks and brown rice from collections of cultivated species of rice using the effect of bacterial growth measured by the MTS assay as phenotype data. SNPs significantly associated with the phenotype at a − log10(*p*) value greater than 5 were detected in the phenotype data of 80% MeOH rice hull extract, acetone brown rice extract, and 80% MeOH brown rice extract (Fig. 7a–c, Additional file 2: Fig. S1). Among these SNPs, a total of 499 SNPs were selected based on filters of FDR < 0.05 and significant associations below the *p*-values, and these SNPs were located in 42 linkage disequilibrium (LD) blocks (Table 3). Next, we examined the effect of each haplotype on the bacterial growth at five SNPs with the lowest *p*-values in an LD block, we found significant differences in bacterial growth among haplotypes for four of the five SNPs (Fig. 7 d–g). The linkage disequilibrium regions where the four SNPs locate cover the 43 kb, 206 kb, 161 kb, and 28 kb regions on chromosomes 3, 4, 7, and 9, respectively. This suggests that there are single or multiple genetic factors affecting bacterial growth by seed extracts in these regions. Thus, the quantitative measurement of antibacterial activity using the MTS method can be used to approach the genetic basis of the production of seed-derived chemicals that affect bacterial growth using GWAS and possibly other methods such as biparental QTL analysis.

**Table 3:** List of significantly associated SNPs in GWAS using antimicrobial activities in the 107 accessions of landraces from WRC and JRC

LD block number	Associated SNP number	SNP with highest association in the LD	CHR	Coordinate	− log10(*p*)
1	1	Yes	1	18,005,951	5.5737002
2	2		1	27,878,359	5.8942855
2	3		1	27,880,379	5.8942855
2	4		1	27,880,453	5.8942855
2	5		1	27,881,781	5.8942855
2	6		1	27,881,834	5.8942855
2	7		1	27,882,081	5.8942855
2	8		1	27,882,248	5.8942855
2	9		1	27,882,325	5.8942855
2	10		1	27,883,352	5.7644463
2	11		1	27,885,038	6.0783968
2	12		1	27,886,765	6.1436155
2	13		1	27,886,937	6.0783968
2	14		1	27,887,170	6.0783968
2	15		1	27,887,655	6.0783968
2	16		1	27,887,678	6.0783968
2	17		1	27,887,813	6.0783968
2	18		1	27,887,821	6.0783968
2	19		1	27,887,894	6.0783968
2	20		1	27,889,339	6.0783968
2	21		1	27,889,390	6.0783968
2	22		1	27,890,608	6.0092839
2	23		1	27,891,417	5.8942855
2	24		1	27,891,708	6.0783968
2	25		1	27,892,211	5.8573603
2	26		1	27,893,209	5.8942855
2	27		1	27,893,722	5.8573603
2	28		1	27,893,863	5.8942855
2	29		1	27,894,089	5.8942855
2	30		1	27,894,427	5.8573603
2	31		1	27,894,659	5.8942855
2	32		1	27,894,833	5.8942855
2	33		1	27,894,855	5.8942855
2	34		1	27,895,567	5.8942855
2	35		1	27,895,950	5.8942855
2	36		1	27,897,746	5.8942855
2	37		1	27,897,749	5.8942855
2	38		1	27,905,911	5.8942855
2	39		1	27,905,926	5.8942855
2	40		1	27,906,578	5.8942855
2	41		1	27,906,583	5.8705357
2	42		1	27,907,200	5.8942855
2	43		1	27,910,437	5.8942855
2	44		1	27,916,341	5.8942855
2	45		1	27,921,389	5.8942855
2	46		1	27,921,462	5.8942855
2	47		1	27,921,505	5.8942855
2	48		1	27,923,341	5.8942855
2	49		1	27,925,445	5.8942855
2	50		1	27,925,448	5.8942855
2	51		1	27,929,471	5.8942855
2	52		1	27,929,485	5.8942855
2	53		1	27,936,334	5.8921459
2	54		1	27,936,425	5.8921459
2	55		1	27,936,624	5.8921459
2	56		1	27,938,041	5.8921459
2	57		1	27,938,359	5.5031395
2	58		1	27,939,515	5.8921459
2	59		1	27,949,632	5.8942855
2	60		1	27,950,033	5.8942855
2	61	Yes	1	27,950,388	6.1916607
2	62		1	27,951,516	5.8942855
2	63		1	27,951,520	5.5143779
2	64		1	27,951,622	5.8942855
2	65		1	27,951,679	5.8942855
2	66		1	27,951,730	5.8942855
2	67		1	27,951,846	5.8942855
2	68		1	27,953,717	5.8921459
2	69		1	27,953,871	5.8921459
2	70		1	27,953,994	5.8921459
2	71		1	27,954,243	5.8921459
2	72		1	27,954,363	5.8921459
2	73		1	27,954,387	5.8921459
2	74		1	27,954,496	5.8921459
2	75		1	27,954,743	5.8921459
2	76		1	27,955,180	5.8921459
2	77		1	27,955,491	5.8921459
2	78		1	27,956,227	5.8921459
2	79		1	27,956,293	5.8921459
2	80		1	27,958,781	5.8921459
2	81		1	27,961,451	5.8921459
2	82		1	27,961,827	5.793417
2	83		1	27,961,979	5.8921459
2	84		1	27,962,184	5.8921459
2	85		1	27,964,204	5.8921459
2	86		1	27,965,101	5.8921459
2	87		1	27,965,327	5.8921459
2	88		1	27,966,621	5.8921459
2	89		1	27,967,087	5.8921459
2	90		1	27,968,585	5.8921459
2	91		1	27,969,991	5.8921459
2	92		1	27,970,071	5.8921459
2	93		1	27,972,673	5.8921459
2	94		1	27,973,326	5.8921459
2	95		1	27,973,533	5.8921459
2	96		1	27,980,057	5.8921459
2	97		1	27,980,136	5.8921459
2	98		1	27,981,282	5.8921459
2	99		1	27,981,536	5.8921459
3	100	Yes	2	5,087,458	5.0276583
3	101		2	5,102,106	4.7984936
3	102		2	5,102,126	4.7984936
4	103		2	24,844,697	5.0179777
4	104		2	24,880,854	4.9434382
4	105		2	24,882,717	4.9434382
4	106	Yes	2	24,884,774	5.2144851
4	107		2	24,915,038	4.9434382
4	108		2	24,915,059	4.9434382
5	109	Yes	2	31,700,860	4.9846402
5	110		2	31,704,278	4.9846402
5	111		2	31,704,685	4.9846402
5	112		2	31,706,449	4.9846402
5	113		2	31,714,007	4.9846402
5	114		2	31,714,952	4.9846402
5	115		2	31,722,293	4.9846402
5	116		2	31,728,246	4.9846402
5	117		2	31,728,257	4.9846402
5	118		2	31,734,360	4.9846402
5	119		2	31,735,654	4.9846402
5	120		2	31,736,321	4.9846402
5	121		2	31,737,988	4.9846402
5	122		2	31,740,565	4.9846402
5	123		2	31,742,890	4.9846402
5	124		2	31,746,201	4.9846402
5	125		2	31,746,203	4.9846402
5	126		2	31,757,993	4.9846402
5	127		2	31,762,177	4.9846402
5	128		2	31,762,204	4.9846402
5	129		2	31,773,582	4.9846402
6	130		3	23,599,267	5.4185624
6	131		3	23,602,834	5.4185624
6	132		3	23,602,836	5.4185624
6	133		3	23,603,512	5.4185624
6	134		3	23,604,571	4.9748581
6	135		3	23,604,576	4.9748581
6	136		3	23,605,736	5.4185624
6	137	Yes	3	23,619,690	6.2848578
7	138		3	23,703,226	5.5702154
7	139		3	23,703,283	5.5702154
7	140		3	23,705,154	6.9193735
7	141		3	23,705,351	6.9085089
7	142		3	23,705,455	8.5849765
7	143		3	23,705,458	7.3314836
7	144		3	23,705,491	7.3314836
7	145		3	23,705,652	7.1631128
7	146		3	23,705,694	7.9566377
7	147		3	23,705,695	7.0222627
7	148		3	23,706,631	8.1004531
7	149		3	23,706,709	8.3545679
7	150		3	23,707,032	5.5702154
7	151		3	23,707,299	5.5702154
7	152		3	23,708,043	8.1004531
7	153		3	23,708,062	8.3545679
7	154		3	23,708,620	5.5702154
7	155		3	23,710,275	8.1004531
7	156		3	23,710,359	8.1004531
7	157		3	23,710,419	5.6737461
7	158		3	23,710,504	6.9193735
7	159		3	23,710,544	8.3545679
7	160		3	23,710,576	5.5702154
7	161		3	23,711,028	6.0668419
7	162		3	23,711,636	8.1004531
7	163		3	23,711,749	8.4396629
7	164		3	23,712,310	8.3545679
7	165		3	23,712,325	8.1004531
7	166		3	23,713,421	8.1004531
7	167		3	23,713,435	5.5702154
7	168		3	23,715,323	7.3879052
7	169	Yes	3	23,715,931	9.3375444
7	170		3	23,716,016	9.1989532
7	171		3	23,716,745	5.8389016
7	172		3	23,716,746	8.3650091
7	173		3	23,716,755	8.1796015
7	174		3	23,720,991	8.1004531
7	175		3	23,721,260	8.3545679
7	176		3	23,722,660	5.5702154
7	177		3	23,722,804	5.5702154
7	178		3	23,723,013	5.5702154
7	179		3	23,724,952	5.5702154
7	180		3	23,725,905	8.1004531
7	181		3	23,725,960	8.3545679
7	182		3	23,726,052	8.3545679
7	183		3	23,726,144	5.5702154
7	184		3	23,726,921	5.5702154
7	185		3	23,727,594	5.4542477
7	186		3	23,727,607	5.5702154
7	187		3	23,729,521	5.5702154
7	188		3	23,731,153	5.5702154
7	189		3	23,731,242	5.5702154
8	190	Yes	3	23,899,609	5.1917485
9	191	Yes	3	24,212,274	4.796233
10	192		3	24,446,000	5.7211321
10	193		3	24,446,501	5.7211321
10	194		3	24,447,171	5.7211321
10	195	Yes	3	24,447,470	6.2612987
10	196		3	24,447,579	6.0825733
11	197	Yes	3	27,810,949	5.0142548
12	198	Yes	3	33,333,544	5.3214088
13	199		3	34,199,211	6.3678668
13	200		3	34,200,889	6.3678668
13	201		3	34,202,851	6.3678668
13	202		3	34,207,764	6.292728
13	203		3	34,210,099	6.3678668
13	204		3	34,210,389	6.3678668
13	205		3	34,210,674	6.2748973
13	206		3	34,210,690	6.2748973
13	207		3	34,210,696	6.2748973
13	208		3	34,210,772	6.3678668
13	209		3	34,215,449	6.0992343
13	210		3	34,217,835	6.3678668
13	211		3	34,218,523	6.3678668
13	212		3	34,219,544	6.3678668
13	213		3	34,229,546	6.3678668
13	214		3	34,255,810	6.3678668
13	215		3	34,275,277	6.3678668
13	216		3	34,278,017	6.3678668
13	217		3	34,278,817	6.3678668
13	218		3	34,283,349	6.3678668
13	219	Yes	3	34,373,584	6.4472466
13	220		3	34,382,599	6.3678668
13	221		3	34,385,992	6.3678668
13	222		3	34,386,019	6.3678668
13	223		3	34,389,605	6.3678668
14	224	Yes	4	2,856,477	5.1343572
15	225		4	4,868,339	5.6474895
15	226		4	4,914,935	5.5280193
15	227		4	5,005,023	4.9696027
15	228		4	5,009,730	5.9454252
15	229		4	5,010,358	5.9454252
15	230		4	5,011,336	4.8936051
15	231		4	5,011,658	5.9454252
15	232		4	5,013,003	5.9454252
15	233		4	5,013,314	6.0225279
15	234		4	5,022,565	6.2031621
15	235		4	5,025,728	6.2059583
15	236		4	5,026,779	6.2059583
15	237		4	5,027,101	6.2059583
15	238		4	5,027,514	6.2059583
15	239		4	5,028,468	6.2059583
15	240		4	5,029,403	6.2059583
15	241		4	5,031,991	6.2059583
15	242		4	5,033,295	6.2059583
15	243		4	5,033,475	5.4984068
15	244		4	5,033,545	6.2059583
15	245		4	5,033,552	6.2059583
15	246		4	5,033,624	6.2059583
15	247		4	5,034,748	5.9848918
15	248		4	5,035,576	6.2059583
15	249		4	5,035,806	6.2059583
15	250		4	5,037,743	6.2059583
15	251		4	5,037,825	6.2059583
15	252	Yes	4	5,038,844	6.310407
15	253		4	5,039,716	6.2961758
15	254		4	5,045,202	6.2059583
15	255		4	5,096,994	6.2059583
15	256		4	5,100,290	5.6871248
15	257		4	5,128,610	4.9540905
15	258		4	5,128,681	4.9540905
15	259		4	5,134,602	4.8384224
16	260	Yes	4	30,886,560	5.7629089
16	261		4	30,886,994	5.7629089
16	262		4	30,920,022	4.8718593
16	263		4	30,926,071	5.7629089
16	264		4	30,945,182	4.8718593
16	265		4	30,948,864	5.7629089
16	266		4	30,964,390	4.8718593
16	267		4	30,965,051	4.8718593
16	268		4	30,965,344	5.7629089
16	269		4	30,967,249	4.8718593
16	270		4	30,973,636	4.8718593
17	271	Yes	4	31,674,325	8.8057355
18	272	Yes	4	34,786,157	4.8937411
19	273	Yes	6	4,785,418	5.60333
20	274	Yes	6	7,519,840	5.4449539
21	275	Yes	6	25,598,090	6.1028625
22	276		7	264,621	5.3368175
22	277	Yes	7	284,003	5.5136829
22	278		7	291,471	5.432903
23	279		7	6,063,531	6.1889212
23	280	Yes	7	6,064,303	7.2457358
23	281		7	6,064,370	7.2457358
23	282		7	6,065,094	7.2457358
23	283		7	6,065,873	7.2457358
23	284		7	6,066,571	7.2457358
23	285		7	6,067,391	7.2457358
23	286		7	6,070,349	7.2457358
23	287		7	6,070,358	7.2457358
23	288		7	6,070,366	7.2457358
23	289		7	6,070,418	7.2457358
23	290		7	6,070,679	7.2457358
23	291		7	6,084,536	7.2457358
23	292		7	6,084,550	7.2457358
23	293		7	6,085,652	5.2117538
23	294		7	6,085,690	7.2457358
23	295		7	6,085,742	5.2117538
23	296		7	6,085,749	5.2117538
23	297		7	6,085,763	7.2457358
23	298		7	6,085,834	5.2117538
23	299		7	6,086,044	5.2117538
23	300		7	6,086,074	7.2457358
23	301		7	6,086,334	5.2117538
23	302		7	6,086,414	7.2457358
23	303		7	6,086,726	5.2117538
23	304		7	6,086,998	7.2457358
23	305		7	6,087,022	7.2457358
23	306		7	6,087,080	7.2457358
23	307		7	6,087,198	7.2457358
23	308		7	6,087,516	7.2457358
23	309		7	6,087,526	6.153898
23	310		7	6,087,573	7.2457358
23	311		7	6,087,600	7.2457358
23	312		7	6,087,643	7.2457358
23	313		7	6,087,826	7.2457358
23	314		7	6,087,834	7.2457358
23	315		7	6,088,030	7.2457358
23	316		7	6,088,069	7.2457358
23	317		7	6,088,359	5.2117538
23	318		7	6,090,769	4.768021
23	319		7	6,093,126	5.8051805
23	320		7	6,093,580	5.2117538
23	321		7	6,093,671	5.2117538
23	322		7	6,093,908	5.2117538
23	323		7	6,094,396	5.8051805
23	324		7	6,094,482	5.8051805
23	325		7	6,094,801	5.8051805
23	326		7	6,094,895	5.8051805
23	327		7	6,094,937	5.8051805
23	328		7	6,094,964	5.8051805
23	329		7	6,095,324	5.8051805
23	330		7	6,095,356	5.8051805
23	331		7	6,095,484	5.4227381
23	332		7	6,095,853	5.3338753
23	333		7	6,096,273	5.8051805
23	334		7	6,096,485	5.8051805
23	335		7	6,096,497	4.9147451
23	336		7	6,096,521	5.8051805
23	337		7	6,096,547	5.8051805
23	338		7	6,096,809	5.9043426
23	339		7	6,098,286	5.9043426
23	340		7	6,098,386	5.8051805
23	341		7	6,098,581	5.8051805
23	342		7	6,098,852	5.8051805
23	343		7	6,099,226	5.8051805
23	344		7	6,099,258	5.8051805
23	345		7	6,099,332	5.9043426
23	346		7	6,099,594	5.9043426
23	347		7	6,099,686	5.9043426
23	348		7	6,099,725	5.9043426
23	349		7	6,100,039	5.9043426
23	350		7	6,100,437	5.9043426
23	351		7	6,100,990	5.8051805
23	352		7	6,101,657	5.8051805
23	353		7	6,102,160	5.7175365
23	354		7	6,102,520	5.8051805
23	355		7	6,102,592	4.8790972
23	356		7	6,102,638	4.8790972
23	357		7	6,102,693	4.8790972
23	358		7	6,102,735	4.8790972
23	359		7	6,102,903	4.8790972
23	360		7	6,103,174	5.8051805
23	361		7	6,103,463	4.8790972
23	362		7	6,103,806	4.8790972
23	363		7	6,104,116	4.8790972
23	364		7	6,104,228	5.8051805
23	365		7	6,104,370	5.8395915
23	366		7	6,104,431	4.8790972
23	367		7	6,104,667	4.8790972
23	368		7	6,104,785	4.8790972
23	369		7	6,105,088	4.8790972
23	370		7	6,105,180	5.8051805
23	371		7	6,105,212	5.8051805
23	372		7	6,105,458	4.8790972
23	373		7	6,105,479	5.8051805
23	374		7	6,105,480	5.8051805
23	375		7	6,105,492	5.8051805
23	376		7	6,106,134	6.9801363
23	377		7	6,108,273	4.8790972
23	378		7	6,108,355	5.8051805
23	379		7	6,108,654	5.8051805
23	380		7	6,109,115	4.8790972
23	381		7	6,109,415	5.8051805
23	382		7	6,109,443	5.8395915
23	383		7	6,109,481	5.8051805
23	384		7	6,109,866	5.8051805
23	385		7	6,109,868	6.9801363
23	386		7	6,109,916	4.8790972
23	387		7	6,110,331	4.8790972
23	388		7	6,111,183	4.8790972
23	389		7	6,111,369	4.8790972
23	390		7	6,111,870	4.8790972
23	391		7	6,112,133	5.8051805
23	392		7	6,112,824	5.8051805
23	393		7	6,113,492	4.8790972
23	394		7	6,113,568	4.8790972
23	395		7	6,114,786	4.8790972
23	396		7	6,116,319	4.788826
23	397		7	6,116,607	4.788826
23	398		7	6,116,670	4.788826
23	399		7	6,117,084	4.788826
23	400		7	6,117,920	4.788826
23	401		7	6,117,951	4.788826
23	402		7	6,118,057	4.788826
23	403		7	6,119,445	4.788826
23	404		7	6,119,850	4.788826
23	405		7	6,120,052	4.788826
23	406		7	6,122,777	4.7337213
23	407		7	6,130,228	4.788826
23	408		7	6,130,337	4.788826
23	409		7	6,132,970	4.788826
23	410		7	6,133,017	4.788826
23	411		7	6,133,379	4.788826
23	412		7	6,169,691	4.8181564
23	413		7	6,199,788	5.0076188
23	414		7	6,199,806	5.0076188
24	415		7	6,655,446	4.9209635
24	416	Yes	7	6,670,111	5.4666983
25	417	Yes	7	15,950,927	5.0870132
25	418		7	15,951,213	5.0870132
25	419		7	15,951,648	5.0870132
25	420		7	15,952,726	5.0870132
26	421	Yes	8	244,512	4.7358436
26	422		8	265,949	4.7358436
26	423		8	272,578	4.7358436
26	424		8	272,721	4.7358436
27	425	Yes	8	25,340,714	5.2533736
28	426	Yes	8	25,532,049	5.2987689
29	427	Yes	9	7,166,403	9.2837879
29	428		9	7,168,080	6.1269237
29	429		9	7,168,949	6.1269237
30	430	Yes	9	7,998,198	4.718603
31	431		9	9,668,007	5.6750183
31	432	Yes	9	9,668,585	5.7282745
31	433		9	9,669,315	5.7282745
31	434		9	9,670,051	5.7282745
31	435		9	9,670,151	5.7282745
31	436		9	9,670,373	5.7282745
32	437		9	9,778,486	5.7282745
32	438		9	9,778,556	5.7282745
32	439		9	9,778,722	4.9079815
32	440		9	9,778,764	5.7282745
32	441		9	9,778,873	4.9079815
32	442		9	9,778,945	4.9079815
32	443		9	9,779,063	5.7282745
32	444		9	9,779,075	5.7282745
32	445		9	9,779,123	5.7282745
32	446		9	9,779,138	5.7282745
32	447		9	9,779,269	5.7282745
32	448		9	9,779,315	5.7282745
32	449		9	9,779,351	4.9079815
32	450		9	9,779,353	4.9079815
32	451		9	9,779,376	5.7282745
32	452		9	9,779,470	5.7282745
32	453		9	9,779,619	4.9079815
32	454		9	9,779,834	4.9079815
32	455		9	9,779,839	5.7282745
32	456		9	9,779,846	4.9079815
32	457		9	9,779,938	4.9079815
32	458		9	9,779,996	5.7282745
32	459		9	9,780,618	5.7282745
32	460		9	9,780,698	5.7282745
32	461		9	9,782,756	5.7664962
32	462		9	9,783,743	5.7282745
32	463		9	9,784,795	6.0492653
32	464		9	9,784,952	5.7282745
32	465		9	9,785,403	5.9725688
32	466		9	9,785,621	5.9725688
32	467		9	9,785,899	5.9725688
32	468	Yes	9	9,785,988	6.698189
32	469		9	9,786,291	5.9725688
32	470		9	9,788,095	5.7282745
32	471		9	9,788,526	5.7282745
33	472		9	9,925,206	5.6003263
33	473		9	9,925,891	5.527741
33	474		9	9,926,313	5.1139043
33	475		9	9,926,854	5.6642413
33	476		9	9,927,170	5.3699792
33	477		9	9,927,826	5.3652004
33	478	Yes	9	9,928,848	6.1659864
33	479		9	9,929,137	5.6642413
33	480		9	9,935,223	5.6642413
33	481		9	9,936,891	5.0602412
33	482		9	9,939,176	5.6642413
33	483		9	9,939,470	5.6642413
33	484		9	9,941,053	5.0602412
33	485		9	9,941,753	5.6642413
33	486		9	9,941,774	5.6642413
33	487		9	9,942,709	5.6642413
34	488	Yes	9	14,962,678	4.7118402
35	489	Yes	9	15,900,636	4.7943897
36	490		9	20,146,598	4.9416879
36	491		9	20,155,748	4.8397716
36	492	Yes	9	20,209,460	5.0262212
37	493		10	2,057,109	5.453482
37	494	Yes	10	2,057,364	5.4953117
38	495	Yes	10	21,819,222	5.3096533
39	496	Yes	11	21,990,372	4.7907263
40	497	Yes	11	23,007,993	5.0269461
41	498	Yes	12	19,752,661	4.7299037
42	499	Yes	12	25,568,747	4.8310019

### Quantification of Antibacterial Activity in Extracts of Husk and Brown Rice from Collections of Wild *Oryza* Species

**Fig. 8:**
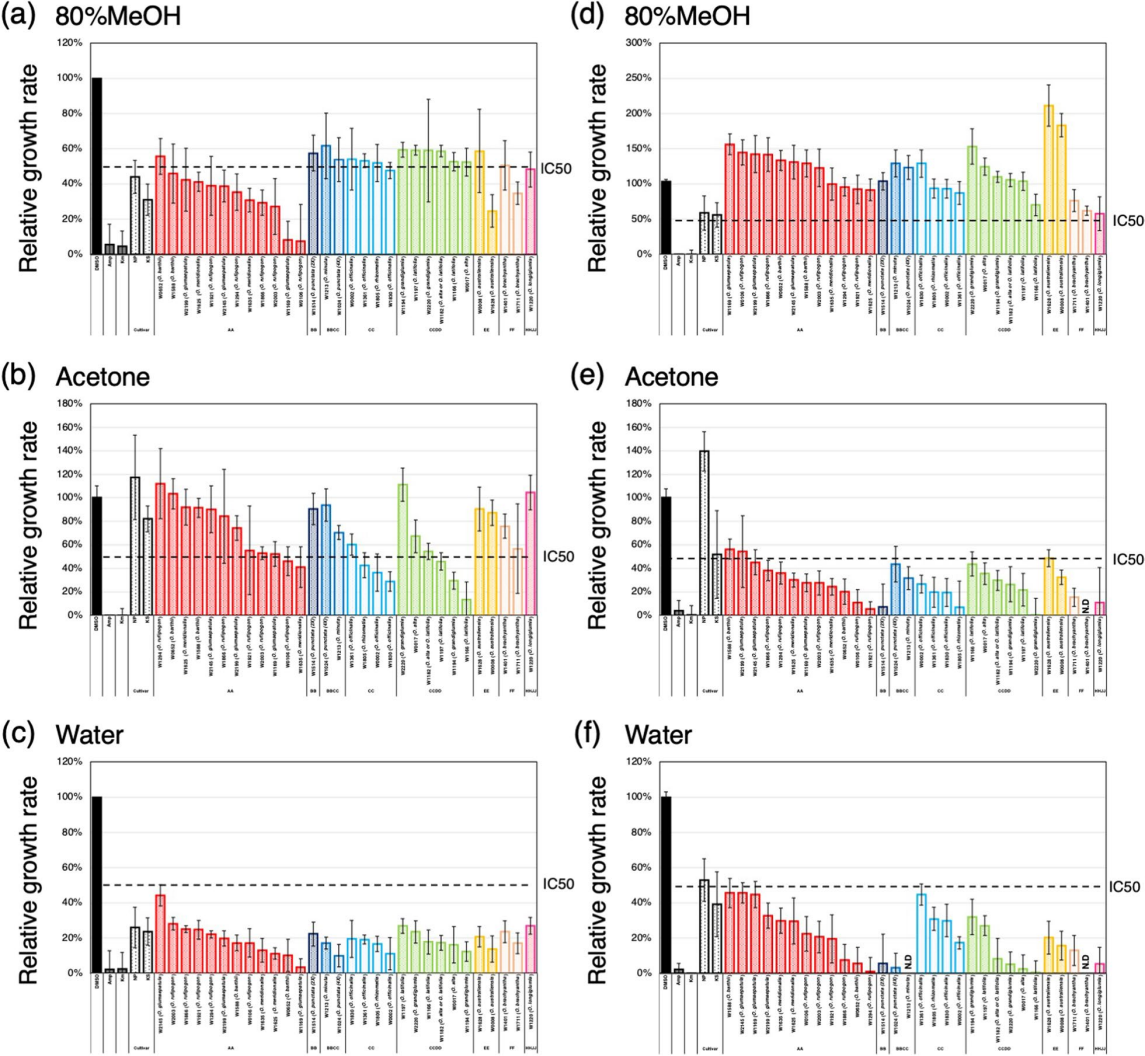
Antibacterial activities of rice seed crude extracts from wild *Oryza* species measured by MTS assay. Antibacterial activities of husk (**a**–**c**) and brown rice (**d**–**f**) crude extracts were measured. Two cultivated rice, Kasalath (KS) and Nipponbare (NB), were added as a control. Solvents for extraction were 80% MeOH (**a**, **d**) acetone (**b**, **e**), and sterilized water (**c**, **f**), respectively. x axis indicates the name of the extracts. y axis indicates relative growth rate of *E. coli*, which represents the antibacterial activity of the sample extracts. DMSO and antibiotics (ampicillin and kanamycin) are negative and positive controls of antibacterial activity. Stock solution of extracts were diluted by the concentration of extracts using either 2.5 seeds (**a**, **c**) or 5 seeds (**b**) for extraction and used for the measurement. All measurements were triplicated. Values indicate means. Error bars indicate standard deviation. N.D. indicates undetectable level of antibacterial activity. IC50 indicates 50% of maximum inhibitory concentration of ampicillin, which is a positive control

In order to understand the diversity of seed defense against environmental microorganisms among wild *Oryza* species, we measured antibacterial activity in extracts of husk and brown rice from 30 accessions covering 15 wild *Oryza* species with 8 genome types by MTS assay (Kurata et al. 2010; Nonomura et al. 2010). As reference, we also measured antibacterial activity in two cultivars, Nipponbare (NP) and Kasalath (KS). 80% MeOH extracts of husks from 15 of the 30 accessions inhibited bacterial growth to less than 50% and extracts from two of them inhibited to less than 20% (Fig. 8a). Acetone extracts of husks from 8 of the 30 accessions inhibited growth to less than 50%, with extracts from one accession inhibited growth to less than 20% (Fig. 8b). Water extracts of husk from all 30 accessions inhibited growth to less than 50%, with extracts from 10 of them inhibited growth to less than 20% (Fig. 8c). 80% MeOH extracts of brown rice from none of the 30 accessions inhibited bacterial growth to less than 50% (Fig. 8d). Acetone extracts of brown rice from most of the 30 accessions inhibited growth to less than 50%, with extracts from 10 accessions inhibited growth to less than 20% (Fig. 8e). Water extracts of brown rice from all 30 accessions inhibited growth to less than 50%, with extracts from 16 of them inhibited growth to less than 20% (Fig. 8f). Surprisingly, the growth-promoting effect which was often observed in brown rice acetone extracts from cultivated species was barely observed in wild *Oryza*, instead, brown rice 80% MeOH extracts from several wild *Oryza* promoted bacterial growth compared to cultivated species. Another important finding in the analysis using wild *Oryza* is that there are several accessions whose seed extracts from two or more solvents show strong bacterial growth inhibition. For example, both water and 80% MeOH extracts of husks of W1169 (*O. glumaepatula*) showed strong bacterial growth inhibition. Similarly, brown rice extracts with two different solvents from W1166 (*O. latifolia*) and W1921 (*O. rufipogon*) showed strong growth inhibition. In addition, brown rice extracts from all three solvents in W1401 (*O. brachyantha*) showed very strong (undetectable levels of bacterial growth) or relatively strong growth inhibition. These results suggest that some of wild *Oryza* accumulate multiple growth inhibitory substances in grains. Overall, the MTS assay is also applicable to assess the effects on bacterial growth by the seed extracts prepared from wild *Oryza*.

## Discussion

Seeds interact with a variety of microorganisms in the environment, and microbial infection and proliferation can cause seed rot and non-germination (Mizobuchi et al. 2018; Fuerst et al. 2014, 2018). Although the interactions between plants and the microorganisms that infect them have been studied mainly in the aboveground plant body and rhizosphere, much is not known about the interactions between seeds and microorganisms, especially the defense mechanisms of seeds against microorganisms. It is known that defense against microorganisms in the environment is important for seeds to germinate. One of the defense mechanisms of seeds is chemical defense by antimicrobial compounds. Rice produces antimicrobial compounds such as momilactones are known to accumulate in the husks of cultivated rice (Kato et al. 1973; Minh et al. 2018). This suggests that momilactones may contribute to the chemical defense of rice seeds. On the other hand, much remains unknown about whether compounds other than momilactones contribute to rice seed chemical defense. To address these issues, we established two bioassay systems suitable for simple and rapid determination of seed-derived antibacterial activity in a variety of materials, including landraces and wild *Oryza* species.

The results of seed antibacterial activity measurements using cultivated rice accessions by the disk diffusion and colorimetric quantification methods established in this study showed generally consistent trends, but overall, the colorimetric method with MTS was able to detect antibacterial activity in a wider range of accessions (Fig. 5a–c). The growth inhibition circle of the disk diffusion method is often affected by the extent of the diffusion of compounds into the medium. Therefore, even if the crude extract contains compounds with antimicrobial activity, the activity may not be detected by the disk diffusion method (Valgas et al. 2007; Bubonja-Sonje et al. 2011). Furthermore, in cases where antimicrobial compounds do not inhibit but slow down the growth of bacteria, it is conceivable that the disk diffusion method may not be able to observe growth inhibition circles. These may be the reasons why antibacterial activity could be detected in more accessions than in the MTS assay.

In this study, the cultivated rice core collections, which cover a wide range of genetic diversity, were used to determine the strength of the seed defense mechanisms against microbial infection. These materials include 28 *indica* and 58 *japonica* lines. Seed antibacterial activity of these subspecies was measured in a crude extract of 80% methanol from rice husks, from which momilactones are efficiently extracted (Lee et al. 1999), by the disk diffusion method, and 42.9% and 37.9% of *indica* and *japonica*, respectively, showed growth inhibition circles (Tables 1, 2). Since it is known that momilactones tend to accumulate more in *japonica* than in *indica* (Kariya et al. 2020), it is likely that other antibacterial compounds are present in addition to momilactones at least in *indica* seeds. In fact, momilactones have been reported to exhibit antibacterial activity against *E. coli* at 400 μg/disc in the disk diffusion assay (Fukuta et al. 2007). However, the amount of momilactones contained in rice husk is extremely small, on the order of ng per hull, and is far less than the concentration that shows a growth inhibitory effect against *E. coli* (Quan et al. 2019a, 2019b; Kakar et al. 2019). This suggests that compounds other than momilactones are involved in the antibacterial activity of the seeds. Furthermore, Minh et al. (2018) focused on rice husk as a material including biochemicals and analyzed the metabolites in rice husks. They found that rice husks of Koshihikari contain momilactones, phenolic acids, phenols, and long-chain fatty acids, and exhibit antimicrobial activity against bacteria. Taken together, it is suggested that several antibacterial compounds other than momilactones may contribute to the antibacterial activity in several cultivated rice varieties used in this study. If momilactones function as antibacterial compounds in seeds, it may be due to the combined action of momilactones and other antibacterial compounds rather than momilactones alone.

Both in cultivated and wild *Oryza* species, antibacterial activities were detected either in husks or brown rice and in both. This suggests that the defense mechanism against microorganisms by seeds may operate both in husks and brown rice and that the defense by the husks and brown rice may function independently. In fact, components of rice bran derived from the outermost layer of brown rice aleurone have been reported to exhibit antibacterial activity against bacteria (Arpan et al. 2013; Castanho et al. 2019; Ferdes et al. 2009).

The GWAS performed in this study revealed several genomic regions that are strongly associated with the inhibition of bacterial growth. Among them, several SNPs showed a strong association with the growth inhibition induced by acetone extracts of brown rice. This suggests that the genetic factors responsible for the production of substances inhibiting bacterial growth, which is contained in brown rice, may be located near these SNPs. Because these SNPs were located in a relatively large linkage disequilibrium region, we could not identify the exact genetic factors through this analysis. In the future, genetic factors can be identified through QTL analysis using the progenies of crossed plants between two cultivars with different haplotypes identified from our GWAS analysis. The simple and high throughput seed antibacterial activity assay developed in this study is a possible significance tool to identify genetic factors related to seed antibacterial activity.

In this study, we comprehensively analyzed the antibacterial activity of seeds from a wide range of wild *Oryza* genetic resources. It is known that some wild *Oryza* accessions do not produce known phytoalexins, such as momilactones (Miyamoto et al. 2016; Kariya et al. 2020). Thus, the wild *Oryza* accessions with strong antibacterial activity could be useful materials for understanding seed defense and interactions with microorganisms and for searching for novel compounds related to these interactions. The quantitative assay of seed antibacterial activity through MTS method developed in this study can be used for analyzing materials with a limited number of seeds, such as wild *Oryza* species because this method can measure antibacterial activity with a limited amount of samples. Therefore, in the future, this method can be applied for GWAS analysis using wild *Oryza* genetic resources and for measurement of antibacterial activity after transformation experiments of wild *Oryza* in the process of identifying loci involved in antibacterial substance production (Shimizu-Sato et al. 2020). It is surprising that the growth-promoting effect seen in the acetone extracts is specific to cultivated species. It is intriguing that, during the process of domestication of rice, cultivated rice strains become to accumulate chemicals with a positive effect on bacterial growth.

## Conclusion

Effectiveness and easiness of the bioassay system we developed in this study was confirmed with Gram-negative bacteria, *E. coli*. There is a possibility that this assay system is applicable to other microorganisms including Gram-positive bacteria and fungi. Furthermore, it can be used to investigate antibacterial activity against seed-decaying microorganisms and vertically transmitted pathogenic microorganisms. The use of this bioassay to search for varieties that exhibit potent seed antibacterial activity is expected to accelerate the breeding of varieties resistant to vertically transmitted seed-borne diseases, and with high seed quality after storage, and advance the identification of novel antibacterial compounds.

## Supplementary Information

**Additional file 1: Table S1**. Weight of husks and brown rice from 10 grains of WRC, JRC and wild *Oryza* used in this study.

**Additional file 2: Figure S1**. Genome-wide association study using antibacterial activities of rice seed crude extracts from 107 accessions of landraces from WRC and JRC as phenotype data. (a–c) Manhattan and Quantile–quantile plots of SNPs associated with antibacterial activity in acetone extracts from husk (a), sterilized water extracts from husk (b), and sterilized water extracts from brown rice (c).

## Data Availability

All datasets are available from the corresponding author on reasonable request.

## Abbreviations

MTS: 3-(4,5-Dimethylthiazol-2-yl)-5-(3-carboxymethoxyphenyl)-2-(4-sulfophenyl)-2H-tetrazolium, inner salt
GWAS: Genome-wide association study

## References

[CR1] Alibi S, Crespo D, Navas J (2021). Plant-derivatives small molecules with antibacterial activity. Antibiotics (Basel).

[CR2] Arpan D, Praveen J, Ajay S (2013). Antibacterial activity of rice bran oil. Recent Res Sci Technol.

[CR3] Atwell BJ, Wang H, Scafaro AP (2014). Could abiotic stress tolerance in wild relatives of rice be used to improve *Oryza sativa*?. Plant Sci.

[CR4] Baltas N, Pakyildiz S, Can Z, Dincer B, Kolayli S (2017). Biochemical properties of partially purified polyphenol oxidase and phenolic compounds of *Prunus spinosa* L. subsp. dasyphylla as measured by HPLC-UV. Int J Food Prop.

[CR5] Bednarek P, Osbourn A (2009). Plant-microbe interactions: chemical diversity in plant defense. Science.

[CR6] Ben-Abu Y, Itsko M (2021). Changes in “natural antibiotic” metabolite composition during tetraploid wheat domestication. Sci Rep.

[CR7] Benjamini Y, Hochberg Y (1995). Controlling the false discovery rate: a practical and powerful approach to multiple testing. J R Stat Soc Ser B (Methodol).

[CR8] Benov L (2021). Improved formazan dissolution for bacterial MTT assay. Microbiol Spectrum.

[CR9] Bradbury PJ, Zhang Z, Kroon DE, Casstevens TM, Ramdoss Y, Buckler ES (2007). TASSEL: software for association mapping of complex traits in diverse samples. Bioinformatics.

[CR10] Bubonja-Sonje M, Giacometti J, Abram M (2011). Antioxidant and antilisterial activity of olive oil, cocoa and rosemary extract polyphenols. Food Chem.

[CR11] Cartwright DW, Langcake P, Pryce RJ, Leworthy DP, Ride JP (1981). Isolation and characterization of two phytoalexins from rice as momilactones A and B. Phytochemistry.

[CR12] Castanho A, Lageiro M, Calhelha RC, Ferreira ICFR, Sokovic M, Cunha LM, Brites C (2019). Exploiting the bioactive properties of γ-oryzanol from bran of different exotic rice varieties. Food Funct.

[CR13] Dalling JW, Davis AS, Schutte BJ, Elizabeth Arnold A (2011). Seed survival in soil: interacting effects of predation, dormancy and the soil microbial community. J Ecol.

[CR14] Dalling JW, Davis AS, Arnold AE, Sarmiento C, Zalamea PC (2020). Extending plant defense theory to seeds. Ann Rev Ecol Evol Syst.

[CR15] DePristo MA, Banks E, Poplin R (2011). A framework for variation discovery and genotyping using next-generation DNA sequencing data. Nat Genet.

[CR16] Ebana K, Kojima Y, Fukuoka S, Nagamine T, Kawase M (2008). Development of mini core collection of Japanese rice landrace. Breed Sci.

[CR17] Eloff JN (1998). A sensitive and quick microplate method to determine the minimal inhibitory concentration of plant extracts for bacteria. Planta Med.

[CR18] Ferdes M, Ungureanu C, Radu N, Chirvase AA (2009). Antimicrobial effect of Monascus purpureus red rice against some bacterial and fungal strains. New Biotechnol.

[CR19] Friedman M (2013). Rice brans, rice bran oils, and rice hulls: composition, food and industrial uses, and bioactivities in humans, animals, and cells. J Agric Food Chem.

[CR20] Fuerst EP, Erson JV, Kennedy AC, Gallagher RS (2011). Induction of polyphenol oxidase activity in dormant wild oat (Avena fatua) seeds and caryopses: a defense response to seed decay fungi. Weed Sci.

[CR21] Fuerst EP, Okubara PA, Erson JV, Morris CF (2014). Polyphenol oxidase as a biochemical seed defense mechanism. Front Plant Sci.

[CR22] Fuerst EP, James MS, Pollard AT, Okubara PA (2018). Defense enzyme responses in dormant wild oat and wheat caryopses challenged with a seed decay pathogen. Front Plant Sci.

[CR23] Fukuta M, Xuan TD, Deba F, Tawata S, Khanh TD, Chung IM (2007). Comparative efficacies in vitro of antibacterial, fungicidal, antioxidant, and herbicidal activities of momilatones A and B. J Plant Interact.

[CR24] Gergerich RC, Dolja VV (2006). Introduction to plant viruses, the invisible foe. Plant Health Inst.

[CR25] Gianinetti A, Finocchiaro F, Maisenti F, Satsap DK, Morcia C, Ghizzoni R, Terzi V (2018). The caryopsis of red-grained rice has enhanced resistance to fungal attack. J Fungi.

[CR26] Grela E, Ząbek A, Grabowiecka A (2015). Interferences in the optimization of the MTT assay for viability estimation of *Proteus mirabilis*. Avicenna J Med Biotechnol.

[CR27] Haase H, Jordan L, Keitel L, Keil C, Mahltig B (2017). Comparison of methods for determining the effectiveness of antibacterial functionalized textiles. PLoS ONE.

[CR28] Ishihara A (2021). Defense mechanisms involving secondary metabolism in the grass family. J Pestic Sci.

[CR29] Ishihara A, Hashimoto Y, Tanaka C, Dubouzet JG, Nakao T, Matsuda F, Nishioka T, Miyagawa H, Wakasa K (2008). The tryptophan pathway is involved in the defense responses of rice against pathogenic infection via serotonin production. Plant J.

[CR30] Izawa T, Shimamoto K (1996). Becoming a model plant: the importance of rice to plant science. Trends Plant Sci.

[CR31] Jacquemin J, Bhatia D, Singh K, Wing RA (2013). The International Oryza Map Alignment Project: development of a genus-wide comparative genomics platform to help solve the 9 billion-people question. Curr Opin Plant Biol.

[CR32] Jeandet P (2018). Structure, chemical analysis, biosynthesis, metabolism, molecular engineering, and biological functions of phytoalexins. Molecules.

[CR33] Jerkovic A, Kriegel AM, Bradner JR, Atwell BJ, Roberts TH, Willows RD (2010). Strategic distribution of protective proteins within bran layers of wheat protects the nutrient-rich endosperm. Plant Physiol.

[CR34] Kajiya-Kanegae H, Ohyanagi H, Ebata T, Tanizawa Y, Onogi A, Sawada Y, Hirai MY, Wang ZX, Han B, Toyoda A (2021). OryzaGenome2.1: database of diverse genotypes in wild Oryza species. Rice.

[CR35] Kakar K, Xuan TD, Quan NV, Wafa IK, Tran HD, Khanh TD, Dat TD (2019). Efficacy of N-Methyl-N-Nitrosourea mutation on physicochemical properties, phytochemicals, and momilactones A and B in rice. Sustainability (Switzerland).

[CR36] Kariya K, Murata K, Kokubo Y, Ube N, Ueno K, Yabuta Y, Teraishi M, Okumoto Y, Mori N, Ishihara A (2019). Variation of diterpenoid phytoalexin oryzalexin A production in cultivated and wild rice. Phytochemistry.

[CR37] Kariya K, Ube N, Ueno M, Teraishi M, Okumoto Y, Mori N, Ueno K, Ishihara A (2020). Natural variation of diterpenoid phytoalexins in cultivated and wild rice species. Phytochemistry.

[CR38] Kato T, Kabuto C, Sasaki N, Tsunagawa M, Aizawa H, Fujita K, Kato Y, Kitahara Y (1973). Momilactones, growth inhibitors from rice, *Oryza sativa* L. Tetrahedron Lett.

[CR39] Kawahara Y, de la Bastide M, Hamilton JP, Kanamori H, McCombie WR, Ouyang S, Schwartz DC, Tanaka T, Wu J, Zhou S, Childs KL, Davidson RM, Lin H, Quesada-Ocampo L, Vaillancourt B, Sakai H, Lee SS, Kim J, Numa H, Itoh T, Buell CR, Matsumoto T (2013). Improvement of the Oryza sativa Nipponbare reference genome using next generation sequence and optical map data. Rice (N Y).

[CR40] Kodama O, Miyakawa J, Akatsuka T, Kiyosawa S (1992). Sakuranetin, a flavanone phytoalexin from ultraviolet-irradiated rice leaves. Phytochemistry.

[CR41] Kojima Y, Ebana K, Fukuoka S, Nagamine T, Kawase M (2005). Development of an RFLP-based rice diversity research set of germplasm. Breed Sci.

[CR42] Kurata N, Satoh H, Kitano H, Nagato Y, Endo T, Sato K, Akashi R, Ezura H, Kusaba M, Kobayashi M (2010). NBRP, national bioresource project of Japan and plant bioresource management. Breed Sci.

[CR43] Lee CW, Yoneyama K, Takeuchi Y, Konnai M, Tamagoshi S, Kodama O (1999). Momilactones A and B in rice straw harvested at different growth stages. Biosci Biotechnol Biochem.

[CR44] Li H (2011). A statistical framework for SNP calling, mutation discovery, association mapping and population genetical parameter estimation from sequencing data. Bioinformatics.

[CR45] Liu H, Du Y, Chu H, Shih CH, Wong YW, Wang M, Chu IK, Tao Y, Lo C (2010). Molecular dissection of the pathogen-inducible 3-deoxyanthocyanidin biosynthesis pathway in sorghum. Plant Cell Physiol.

[CR46] Lu F, Ammiraju JSS, Sanyal A, Zhang S, Son R, Chen J, Li G, Sui Y, Song X, Cheng Z (2009). Comparative sequence analysis of MONOCULM1-orthologous regions in 14 Oryza genomes. Proc Natl Acad Sci U S A.

[CR47] McKenna A, Hanna M, Banks E, Sivachenko A, Cibulskis K, Kernytsky A, Garimella K, Altshuler D, Gabriel S, Daly M, DePristo MA (2010). The Genome Analysis Toolkit: a MapReduce framework for analyzing next-generation DNA sequencing data. Genome Res.

[CR48] Minh TN, Xuan TD, Ahmad A, Elzaawely AA, Teschke R, Van TM (2018). Efficacy from different extractions for chemical profile and biological activities of rice husk. Sustainability (Switzerland).

[CR49] Miyamoto K, Fujita M, Shenton MR, Akashi S, Sugawara C, Sakai A, Horie K, Hasegawa M, Kawaide H, Mitsuhashi W (2016). Evolutionary trajectory of phytoalexin biosynthetic gene clusters in rice. Plant J Cell Mol Biol.

[CR50] Mizobuchi R, Fukuoka S, Tsuiki C, Tsushima S, Sato H (2018). Evaluation of major Japanese rice cultivars for resistance to bacterial grain rot caused by Burkholderia glumae and identification of standard cultivars for resistance. Breed Sci.

[CR51] Morimoto N, Ueno K, Teraishi M, Okumoto Y, Mori N, Ishihara A (2018). Induced phenylamide accumulation in response to pathogen infection and hormone treatment in rice (*Oryza sativa*). Biosci Biotechnol Biochem.

[CR52] Morrissey JP, Osbourn AE (1999). Fungal resistance to plant antibiotics as a mechanism of pathogenesis. Microbiol Mol Biol Rev.

[CR53] Murata K, Kitano T, Yoshimoto R, Takata R, Ube N, Ueno K, Ueno M, Yabuta Y, Teraishi M, Holland CK (2020). Natural variation in the expression and catalytic activity of a naringenin 7-O-methyltransferase influences antifungal defenses in diverse rice cultivars. Plant J.

[CR54] Nonomura KI, Morishima H, Miyabayashi T, Yamaki S, Eiguchi M, Kubo T, Kurata N (2010). The wild Oryza collection in National BioResource Project (NBRP) of Japan: History, biodiversity and utility. Breed Sci.

[CR55] Oros G, Kállai Z (2019) Phytoanticipins: the constitutive defense compounds as potential botanical fungicides. In: Bioactive molecules in plant defense: signaling in growth and stress. Springer, pp 179–229. 10.1007/978-3-030-27165-7_11

[CR56] Park HL, Lee SW, Jung KH, Hahn TR, Cho MH (2013). Transcriptomic analysis of UV-treated rice leaves reveals UV-induced phytoalexin biosynthetic pathways and their regulatory networks in rice. Phytochemistry.

[CR57] Park HL, Yoo Y, Hahn TR, Bhoo SH, Lee SW, Cho MH (2014). Antimicrobial activity of UV-induced phenylamides from rice leaves. Molecules.

[CR58] Peters RJ (2006). Uncovering the complex metabolic network underlying diterpenoid phytoalexin biosynthesis in rice and other cereal crop plants. Phytochemistry.

[CR59] Pumirat P, Luplertlop N (2013). The in-vitro antibacterial effect of colored rice crude extracts against staphylococcus aureus associated with skin and soft-tissue infection. J Agric Sci.

[CR60] Quan NV, Thien DD, Khanh TD, Tran HD, Xuan TD (2019). Momilactones A, B, and tricin in rice grain and by-products are potential skin aging inhibitors. Foods.

[CR61] Quan NV, Tran HD, Xuan TD, Ahmad A, Dat TD, Khanh TD, Teschke R (2019). Momilactones A and B are α-amylase and α-glucosidase inhibitors. Molecules.

[CR62] Sato Y, Tsuda K, Yamagata Y, Matsusaka H, Kajiya-Kanegae H, Yoshida Y, Agata A, Ta KN, Shimizu-Sato S, Suzuki T (2021). Collection, preservation and distribution of Oryza genetic resources by the national bioresource project rice (NBRP-rice). Breed Sci.

[CR63] Schmelz EA, Kaplan F, Huffaker A, Dafoe NJ, Vaughan MM, Ni X, Rocca JR, Alborn HT, Teal PE (2011). Identity, regulation, and activity of inducible diterpenoid phytoalexins in maize. Proc Natl Acad Sci U S A.

[CR64] Schmelz EA, Huffaker A, Sims JW, Christensen SA, Lu X, Okada K, Peters RJ (2014). Biosynthesis, elicitation and roles of monocot terpenoid phytoalexins. Plant J.

[CR65] Shimizu-Sato S, Tsuda K, Nosaka-Takahashi M, Suzuki T, Ono S, Ta KN, Yoshida Y, Nonomura KI, Sato Y (2020). Agrobacterium-mediated genetic transformation of wild Oryza species using immature embryos. Rice.

[CR66] Shin J-H, Blay S, McNeney B, Graham J (2006). LDheatmap: an R function for graphical display of pairwise linkage disequilibria between single nucleotide polymorphisms. J Stat Softw Code Snippets.

[CR67] Tanaka N, Shenton M, Kawahara Y, Kumagai M, Sakai H, Kanamori H, Yonemaru J, Fukuoka S, Sugimoto K, Ishimoto M (2020). Whole-genome sequencing of the NARO World Rice Core Collection (WRC) as the basis for diversity and association studies. Plant Cell Physiol.

[CR68] Tanaka N, Shenton M, Kawahara Y, Kumagai M, Sakai H, Kanamori H, Yonemaru JI, Fukuoka S, Sugimoto K, Ishimoto M (2021). Investigation of the genetic diversity of a rice core collection of Japanese landraces using whole-genome sequencing. Plant Cell Physiol.

[CR69] Tsukatani T, Higuchi T, Suenaga H, Akao T, Ishiyama M, Ezoe T, Matsumoto K (2009). Colorimetric microbial viability assay based on reduction of water-soluble tetrazolium salts for antimicrobial susceptibility testing and screening of antimicrobial substances. Anal Biochem.

[CR70] Turner SD (2014) qqman: an R package for visualizing GWAS results using Q-Q and manhattan plots. Biorxiv, 005165. 10.1101/005165

[CR71] Ube N, Katsuyama Y, Kariya K, Tebayashi SI, Sue M, Tohnooka T, Ueno K, Taketa S, Ishihara A (2021). Identification of methoxylchalcones produced in response to CuCl_2_ treatment and pathogen infection in barley. Phytochemistry.

[CR72] Valgas C, De Souza SM, Smânia EFA, Smânia A (2007). Screening methods to determine antibacterial activity of natural products. Braz J Microbiol.

[CR73] VanEtten HD, Mansfield JW, Bailey JA, Farmer EE (1994). Two classes of plant antibiotics: phytoalexins versus “phytoanticipins”. Plant Cell.

[CR74] Yamane H (2013). Biosynthesis of phytoalexins and regulatory mechanisms of it in rice. Biosci Biotechnol Biochem.

[CR75] Zhang C, Dong SS, Xu JY, He WM, Yang TL (2019). PopLDdecay: a fast and effective tool for linkage disequilibrium decay analysis based on variant call format files. Bioinformatics.

