# Supplementary figures and images for: Measurements of Antibacterial Activity of Seed Crude Extracts in Cultivated Rice and Wild Oryza Species
Source: Rice (N Y). 2022 Dec 13;15:63. doi: 10.1186/s12284-022-00610-3 (PMC9748026; doi:10.1186/s12284-022-00610-3)

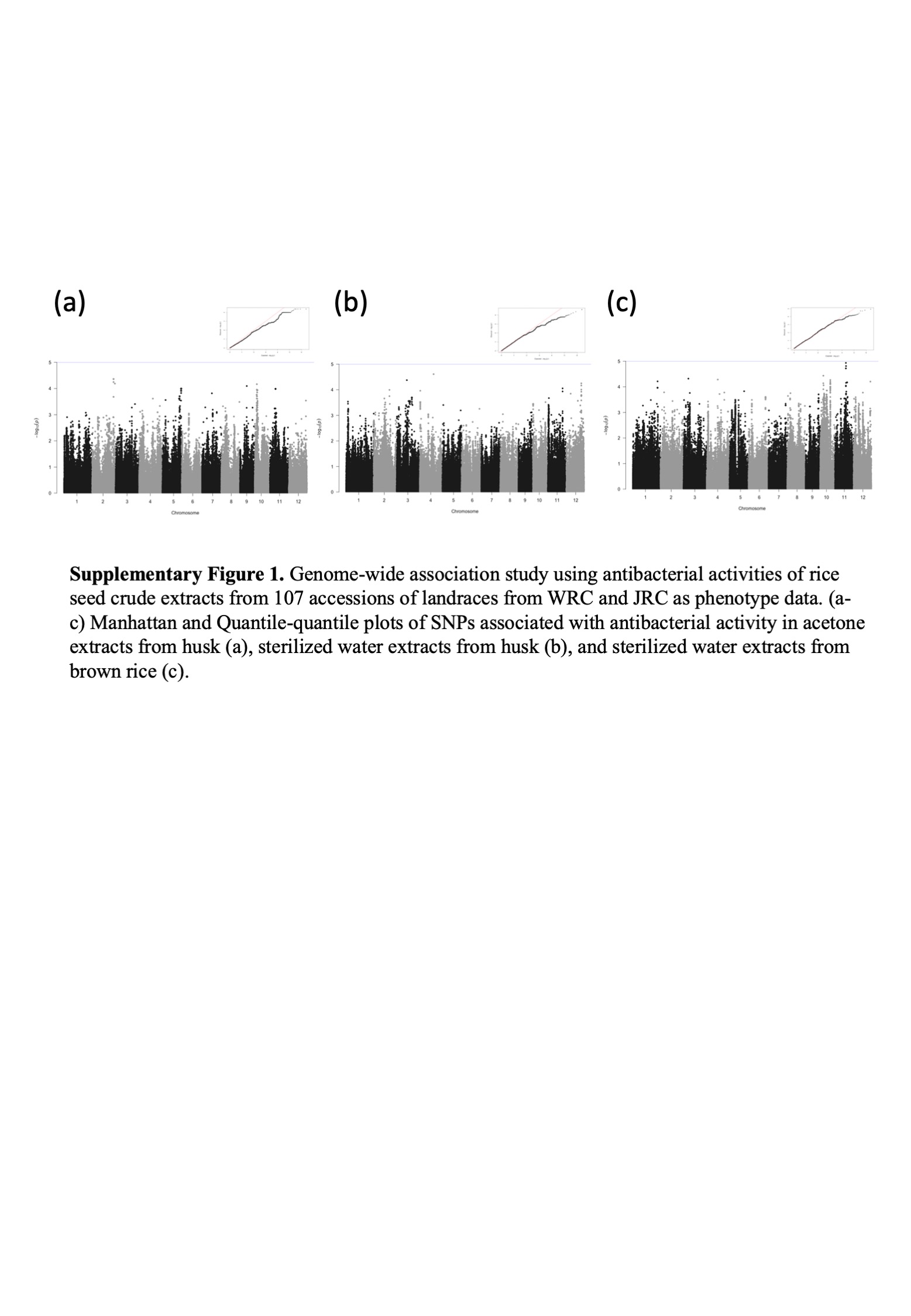

Supplement: Supplementary file 2 — Additional file 2: Figure S1. Genome-wide association study using antibacterial activities of rice seed crude extracts from 107 accessions of landraces from WRC and JRC as phenotype data. (a–c) Manhattan and Quantile–quantile plots of SNPs associated with antibacterial activity in acetone extracts from husk (a), sterilized water extracts from husk (b), and sterilized water extracts from brown rice (c). [file 12284_2022_610_MOESM2_ESM.jpeg]
